# Single microcolony diffusion analysis in *Pseudomonas aeruginosa* biofilms

**DOI:** 10.1038/s41522-019-0107-4

**Published:** 2019-11-08

**Authors:** Jagadish Sankaran, Nicholas J. H. J. Tan, Ka Pui But, Yehuda Cohen, Scott A. Rice, Thorsten Wohland

**Affiliations:** 1 Department of Biological Sciences, National University of Singapore, 117558 Singapore, Singapore; 2 Centre for BioImaging Sciences, National University of Singapore, 117557 Singapore, Singapore; 3 Singapore Centre for Environmental Life Sciences Engineering, Nanyang Technological University, 637551 Singapore, Singapore; 4 School of Biological Sciences, Nanyang Technological University, 637551 Singapore, Singapore; 5 Department of Chemistry, National University of Singapore, 117543 Singapore, Singapore; 6 ithree Institute, University of Technology Sydney, Sydney, Australia

**Keywords:** Antimicrobials, Bacteriology, Biofilms

## Abstract

The influence of the biofilm matrix on molecular diffusion is commonly hypothesized to be responsible for emergent characteristics of biofilms such as nutrient trapping, signal accumulation and antibiotic tolerance. Hence quantifying the molecular diffusion coefficient is important to determine whether there is an influence of biofilm microenvironment on the mobility of molecules. Here, we use single plane illumination microscopy fluorescence correlation spectroscopy (SPIM-FCS) to obtain 3D diffusion coefficient maps with micrometre spatial and millisecond temporal resolution of entire *Pseudomonas aeruginosa* microcolonies. We probed how molecular properties such as size and charge as well as biofilm properties such as microcolony size and depth influence diffusion of fluorescently labelled dextrans inside biofilms. The 2 MDa dextran showed uneven penetration and a reduction in diffusion coefficient suggesting that the biofilm acts as a molecular sieve. Its diffusion coefficient was negatively correlated with the size of the microcolony. Positively charged dextran molecules and positively charged antibiotic tobramycin preferentially partitioned into the biofilm and remained mobile inside the microcolony, albeit with a reduced diffusion coefficient. Lastly, we measured changes of diffusion upon induction of dispersal and detected an increase in diffusion coefficient inside the biofilm before any loss of biomass. Thus, the change in diffusion is a proxy to detect early stages of dispersal. Our work shows that 3D diffusion maps are very sensitive to physiological changes in biofilms, viz. dispersal. However, this study also shows that diffusion, as mediated by the biofilm matrix, does not account for the high level of antibiotic tolerance associated with biofilms.

## Introduction

Biofilms are aggregates of microbes that adhere to biotic or abiotic surfaces encased in a self-produced matrix of extracellular polymeric substances (EPS).^1^ The proximity of cells embedded in the EPS enable intercellular communication and metabolite exchange that underpins the emergence of properties of biofilms that are distinct from free-living planktonic cells.^2^ Some of these emergent properties include signal accumulation, enhanced horizontal gene exchange, differential access to nutrients through sorption to the EPS and an increased tolerance to antimicrobials^3^ and host immune response.^4^ Biofilm formation increases antimicrobial tolerance by several mechanisms, including an increased transmission of resistance between the different organisms within the biofilm due to high population densities and proximity of cells,^5^ sequestration due to interaction with EPS^6^ and the presence of metabolically inactive persister cells that survive antimicrobial treatment.^7^

Differential access to nutrients leads to altered metabolic activity at different regions of a biofilm.^8^ This leads to physiological heterogeneity at multiple spatial scales. There is considerable diversity not only across different microcolonies within a biofilm but also within the different regions of a single microcolony. The architectural and biochemical heterogeneity arises due to chemical gradients,^9,10^ stochastic gene expression^11^ and genetic variation.^12^ This inherent variation is reflected in the emergent properties that arise upon the conversion of planktonic cells into the biofilm state and can be quantified by measuring the abundance of various molecules,^13^ by measuring gene expression levels,^14^ respiratory activity,^15^ stiffness of biofilms^16^ or the diffusion and mobility of molecules within biofilms.^17–28^

**Fig. 1:**
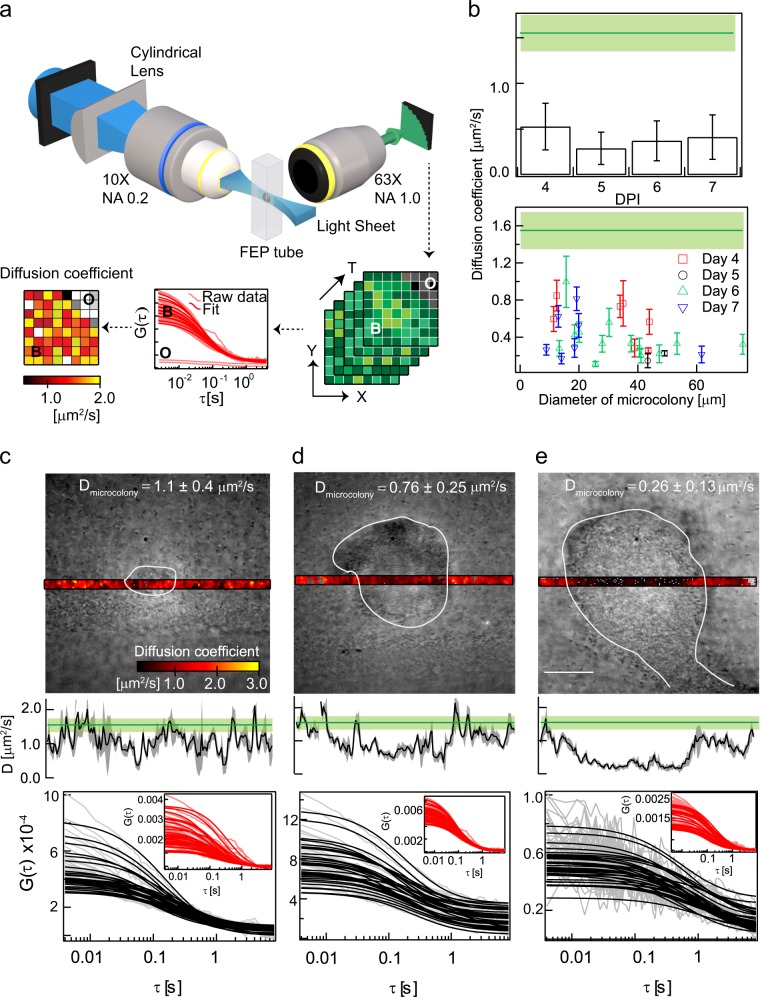
Diffusion of 2 MDa dextran inside biofilms: a schematic of SPIM-FCS is shown in **a**. An EMCCD camera mounted in a light sheet microscope records an image stack. The diffusion coefficients extracted after fitting the calculated autocorrelation functions are displayed as a parametric map. Bright regions in diffusion maps indicate regions of higher mobility when compared to the dim regions, which represent regions of lower mobility. White pixels indicate non-convergence of fits in those regions and hence were not quantified. For the experiments described in this figure, an Andor iXon 897 camera was used as the detector. A plot of the variation in diffusion coefficient of 2 MDa TRITC-dextran with days post inoculation (DPI) is shown in **b** (top). The same data have been replotted according to colony sizes (*N* = 29) in **b** (bottom). The solid green line and the light green box in **b**–**e** is the diffusion coefficient in solution with the corresponding standard deviation. **c**–**e** show the wide-field images overlaid with diffusion maps, spatial diffusion profile and autocorrelation curves of three different microcolonies. The boundaries of the microcolony are shown in white contours. The grey area surrounding the black lines are the associated standard deviation in the spatial diffusion profiles. The autocorrelations inside the microcolony are shown in black while the autocorrelations at the outside are shown in red in the inset. The average diffusion coefficient inside the microcolony is displayed in **c**–**e**. The error bars in **b** show the standard deviation. The scale bar shown in **e** measures 20 µm

The mobility of molecules in biofilms has been quantified using fluorescence recovery after photobleaching,^17,18,22,26,28^ single particle tracking (SPT),^27^ and fluorescence correlation spectroscopy (FCS).^19–21,23–25^ Apart from quantifying the pore size of biofilms^24^ and the mobility of phages in biofilms,^20,21^ FCS studies^29^ have also been used to characterize the role of charge^19,25^ and cell wall hydrophobicity^23^ in determining molecular diffusion in bacterial microcolonies. However, past measurements either determined diffusion only at selected points or were averaged over large volumes of the colonies, thus lacking spatial resolution. To overcome this limitation, we have used here a combination of single plane illumination microscopy (SPIM) and FCS called SPIM-FCS^30,31^ that provides spatially resolved diffusion maps. A typical workflow of SPIM-FCS is shown in Fig. 1a.

SPIM has multiple advantages over the traditionally used confocal microscope. A confocal microscope focuses a laser beam to a small spot and scans this beam over the sample to obtain an image. As the laser beam illuminates all molecules along its path, confocal microscopy requires a pinhole to restrict detection to a small observation volume. Therefore, confocal microscopy sequentially records each point in a sample plane while constantly illuminating the whole sample. This leads to unnecessary photobleaching and phototoxicity. SPIM, on the other hand, illuminates a whole cross-section of the sample using a laser light sheet. An orthogonally placed camera simultaneously records all points. It thus illuminates only those parts of a sample that are recorded and avoids unnecessary photodamage. Hence, in contrast to confocal microscopy, which requires scanning in all three dimensions to reconstruct an image of the entire microcolony, SPIM requires scanning only along the depth of the microcolony. The combination of SPIM with the fast acquisition of FCS allows for determination of diffusion maps in whole cross-sections of biofilms, which can be sequentially recorded at different depths to provide three-dimensional (3D) diffusion maps of whole colonies. This provides contiguous 3D diffusion profiles that can identify regions of different mobility important in biofilm development. Such diffusion maps provide the ability to differentiate gaps in the interstices of neighbouring microcolonies from biomass avoiding erroneous conclusions.^32,33^ SPIM has been used to visualize marine bacteria in their native environment^34^ and to study the population dynamics of artificial^35,36^ and natural^37,38^ communities.^39^ Apart from imaging, SPIM coupled with Transient State Imaging (TRAST) has been used to quantify oxygen abundance in live *Pseudomonas aeruginosa* biofilms.^40^

In this study, we used SPIM-FCS to investigate the influence of molecular size and charge on diffusion within microcolonies of *P. aeruginosa* of different size, age and stage to determine how the biofilm matrix influences molecular mobility and whether the structure of biofilms could be a reason for the reduced efficacy of antibiotics.

## Results

FCS provides two primary read-outs. The first is the width of the autocorrelation function (ACF) from which the diffusion coefficient can be determined. The second is the amplitude of the ACF that is related to the average number of particles seen in the observation volume of a pixel and is thus a measure of concentration. The relation between amplitude and concentration is complex however, and depends on the signal-to-noise ratio as will be discussed later. Here, we use these two parameters to characterize mobility within and structure of the biofilm matrices.

First, we determined the accuracy and precision of SPIM-FCS for diffusion coefficient measurements for dextrans of molecular weights in the range of 4 kDa to 2 MDa and compared those with measurements made using confocal FCS (Supplementary Fig. 1 and Supplementary Notes 1). We next performed SPIM-FCS measurements in alginate beads using fluorescently labelled dextrans of different size and charge to provide a baseline for biofilm measurements (Supplementary Figs 2–7, Supplementary Notes 2–4). Alginate, a component of EPS, can be easily formed into beads and provides a homogeneous environment to test SPIM-FCS. TRITC-dextran molecules of 20 and 150 kDa penetrated into alginate beads and exhibited a retardation in diffusion inside the alginate bead, although diffusion was uniform throughout the beads. In contrast, while the 2 MDa TRITC-dextran penetrated into alginate beads, it showed considerable variation in diffusion coefficient at different positions.

To disentangle size and charge effects, we therefore conducted measurements with 2 MDa TRITC-dextran to investigate sieving effects in *P. aeruginosa* biofilms, while we used 150 kDa dextrans of different charge to investigate the influence of charge on penetration and diffusion. Images of representative *P. aeruginosa* microcolonies fluorescently stained with Con-A-AlexaFluor 488 are shown in Supplementary Fig. 8.

### Diffusion of 2 MDa dextran inside biofilms

TRITC-labelled 2 MDa neutral dextran was added to *P. aeruginosa* biofilms. For biofilms grown for 4–7 days, there was a fourfold reduction in the diffusion coefficient within the biofilm when compared to that in solution (Fig. 1b, top).

We observed a negative correlation between the diffusion coefficient and microcolony size (Fig. 1b, bottom). The Pearson’s linear correlation coefficient was found to be −0.38 (*N* = 29). Due to the heterogeneity in colony sizes on any given day, we did not detect a statistically significant difference in diffusion coefficient across days post inoculation (Fig. 1b, top, one-way ANOVA, *p* = 0.13). Small colonies (diameter < ~40 µm, *N* = 19) had a mean diffusion coefficient of 0.49 ± 0.25 µm^2^ s^−1^, while for larger colonies (diameter > ~40 µm, *N* = 10) the mean diffusion coefficient was 0.28 ± 0.12 µm^2^ s^−1^. Examples of individual colonies ranging from 20 to 70 µm are shown in Fig. 1c, d. While small microcolonies showed uniform diffusion profiles (Fig. 1c, d), microcolonies of ~70 µm diameter, diffusion was reduced in the centre of the large colonies, shown as a trough in the diffusion profile (Fig. 1e).

In addition to the changes in diffusion, the amplitude of the ACFs decreased by about a factor 5 for the smaller colonies (Fig. 1c, d) when comparing the exterior to the interior of the biofilm and almost a factor of 20 for the large colony (Fig. 1e). The correlation curves inside the biofilm were also noisier than those on the outside. The reduction in amplitude and in the signal-to-noise ratio suggests that the concentration inside the biofilm is lower than that of the outside (as described in Supplementary notes S4). This demonstrates that there is a concentration gradient of the 2 MDa TRITC-dextran from the outside to the inside. While qualitatively correct, a quantitative estimation of the concentration cannot be obtained currently due to the lack of methods to estimate the noise levels within individual microcolonies.

### 3D profiles of diffusion coefficients of neutral molecules

**Fig. 2:**
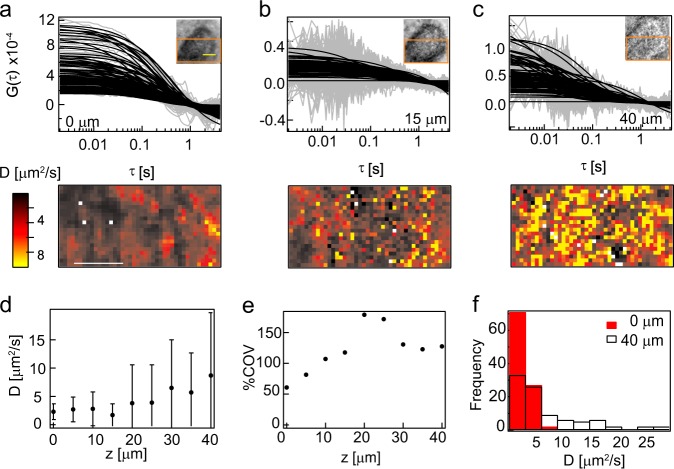
3D profiles of diffusion coefficients of neutral molecules: **a**–**c** show autocorrelation functions, diffusion maps and wide-field images at three different depths of the biofilm that has a thickness of 40 µm. The diffusion map is that of the region shown in orange in the inset of **a** –**c**. In the autocorrelation functions, the raw data are shown in grey while the fitted data are shown in black. The scale bar in yellow in the inset and the diffusion map in **a** is 10 µm. **d** is a plot of the average diffusion coefficient of 2 MDa TRITC-dextran with depth of the biofilm whose wide-field images are shown in Supplementary Fig. 10. The coefficient of variation, which is the ratio of the standard deviation to the mean, is shown in **e**. **f** shows the distribution of diffusion coefficients for the solution and at the base of the biofilm. Supplementary Figure 10 is an accompanying figure to this main figure. The raw and fitted autocorrelation functions along with the diffusion maps at all measured depths is shown in Supplementary Fig. 10. The error bars in **d** show the standard deviation

Given the change in diffusion based on microcolony diameter for 2 MDa TRITC-dextran, we next investigated the relationship between the diffusion coefficient and microcolony depths by analysing diffusion of 2 MDa TRITC-dextran in 11 colonies of varying diameters (20–40 µm). Overall, we saw a change in the signal-to-noise ratio of the ACFs with depth (Fig. 2a–c, top). There was also an increase in heterogeneity in diffusion maps at 15 and 40 µm when compared to 0 µm (Fig. 2a–c, bottom, d). The heterogeneity was quantified by the coefficient of variation (COV ratio of the standard deviation to the mean) of the diffusion coefficient that increases with depth (Fig. 2e). The increased COV was manifested as a wider distribution of diffusion coefficients as can be seen from the diffusion coefficient histograms (Fig. 2f) and is a result of the lower signal-to-noise ratio for these measurements. This implies a decrease in concentration (see Supplementary Note 4) with depth, establishing a molecular gradient.

While the colonies in Fig. 2 and Supplementary Fig. 12 are ~30 µm thick, the colony in Supplementary Fig. 11 is ~14 µm thick. Colonies shown in Fig. 2 and Supplementary Fig. 11 showed considerable changes in COV with depth, while the colony shown in Supplementary Fig. 12 had very little variation in diffusion coefficient, with consistent high signal-to-noise ratio, low COV and narrow distributions of the diffusion coefficient. This implies considerable heterogeneity in depth profiles for the diffusion coefficient and the molecular concentration gradient was not related to the size of the biofilm (as shown in Fig. 2, Supplementary Figs 11 and 12) even when the average diffusion coefficient within the colonies was correlated to overall size.

### Diffusion of charged molecules in biofilms

**Fig. 3:**
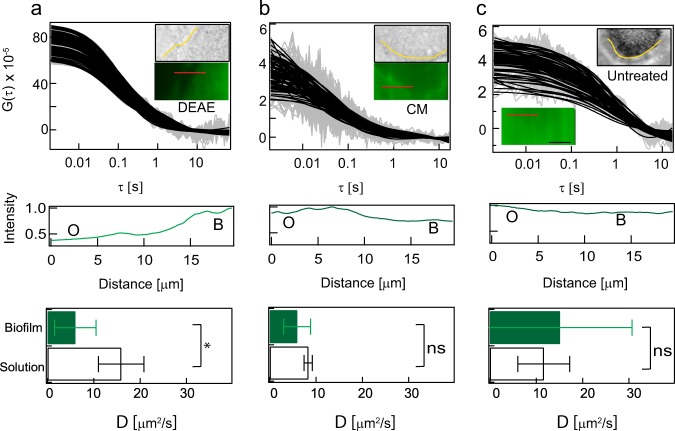
Diffusion of charged molecules in biofilms: **a**–**c** show the wide-field image, fluorescence image and autocorrelation curves when 150 kDa FITC-DEAE-dextran, 150 kDa FITC-CM-dextran and untreated 150 kDa dextran molecules were added to biofilms. The contour of the biofilm is shown in yellow in the wide-field images. The intensity profile along the red line shown in the fluorescence images are shown in the profiles below the autocorrelation curve. O refers to outside while B refers to inside the biofilm. The bar charts show the diffusion coefficients of the fast moving population of positive (*N* = 5), negative (*N* = 3) and neutral (*N* = 8) molecules inside the biofilm and in solution. Only the 150 kDa FITC-DEAE-dextran exhibited a statistically significant reduction (*p* = 0.04) in the diffusion coefficient of the fast moving population inside the biofilm when compared to solution. The raw data and the fitted data are shown in grey and black respectively in **a**–**c**. Individual autocorrelation functions in a representative area of 100 pixels are shown in **a**–**c**. The significance levels were evaluated using a two-sided Wilcoxon rank test. The error bars show the standard deviation. The scale bar in the inset of **c** measures 10 µm

Next, FITC-labelled 150 kDa dextran coupled with positive (diethyl aminoethyl-DEAE; zeta potential = 34.3 ± 3.8 mV) or negative charges (carboxy methyl-CM; zeta potential = −23.5 ± 3.2 mV) were added to biofilms to study how charge affects the diffusion of molecules in microcolonies. When the positively charged 150 kDa FITC-DEAE-dextran (DEAE-dex) was used, it preferentially partitioned into the biofilm (Fig. 3a, inset bottom). The intensity profile along the red line in Fig. 3a shows that the intensity is higher in the biofilm when compared to the exterior. In contrast, the untreated 150 kDa FITC-dextran (zeta potential = −5 ± 3 mV) and 150 kDa FITC-CM-dextran did not preferentially partition into the biofilm (Fig. 3b, c, insets) and the intensities were similar on the outside and inside of the biofilms.

The intensity ratio of the interior of the microcolonies (*N* = 3) to that of the exterior was 3.2 ± 1.0, 0.9 ± 0.1 and 0.8 ± 0.2 for the positively charged, negatively charged and neutral molecules, respectively. In addition, the ACF of 150 kDa FITC-DEAE-dextran had a better signal-to-noise ratio compared to the negative and neutral molecules (Fig. 3a–c). Collectively, these two metrics indicate that the positively charged molecules penetrated with the highest efficacy of the three types of molecules.

The ACF of all three dextrans showed two different diffusion patterns both in solution and in the biofilm and were fitted to a two-component diffusion model (Eq. 3). The slowly diffusing population had diffusion coefficients less than 1 μm^2^ s^−1^, while the fast diffusing population had diffusion coefficient larger than 5 μm^2^ s^−1^. The diffusion coefficients and their fractions are provided in Supplementary Table 7. The presence of two diffusing populations indicates that a certain proportion of probe molecules exist as aggregates in solution and in the biofilm, while the remainder of the probe is present as single molecules.

Only the 150 kDa FITC-DEAE-dextran exhibited a statistically significant reduction (*N* = 5, *p* = 0.04) in the diffusion coefficient of the fast moving population inside the biofilm (6 ± 4.5 µm^2^ s^−1^) when compared to solution (15.9 ± 4.9 µm^2^ s^−1^). The diffusion coefficient of the slow moving population and the fraction of slow moving population was not significantly different between the biofilm and the solution.

### 3D profile of diffusion coefficients of positively charged molecule

**Fig. 4:**
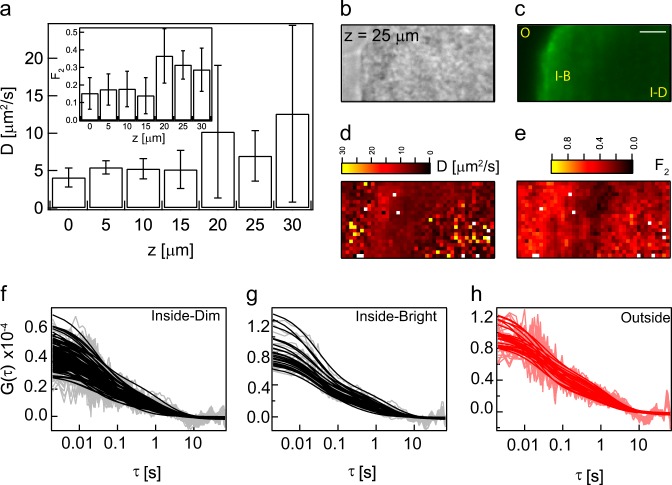
3D profile of diffusion coefficients of positively charged molecule: **a** is a plot of the variation of the diffusion coefficient inside the biofilm with the *z* position when 150 kDa DEAE-dextran was added to the biofilm. The inset shows the fraction of the slow moving particle. Only one plane at *z* = 25 μm is shown here, while the entire *z* stack is shown in Supplementary Fig. 13. **b** and **c** are the wide field and fluorescent images, respectively. O refers to outside, I-B refers to “inside-bright”, I-D refers to “inside-dim”. The faster diffusion coefficient and the fraction of the slow particle (*F*_*2*_) is shown in **d** and **e,** respectively. The autocorrelation curves of the dim region in the interior of the biofilm (I-D) in the fluorescence image is shown in **f**. The autocorrelation curves of bright interior (I-B) region of the biofilm in the fluorescence image is shown in **g**. **h** is a plot of the autocorrelation curves at the outside of the biofilm. The error bars in **a** show the standard deviation. The scale bar in **c** measures 10 µm

Although positively charged molecules penetrated with highest efficiency, the localization and dynamics of DEAE-dextran at different depths of a microcolony was not homogeneous. One specific example is shown in Fig. 4 and Supplementary Fig. 13. As quantified in Supplementary Table 7 earlier, the particles exhibited a two-component diffusion pattern. Inside the microcolony, the heterogeneity in the diffusion map of the faster component increased with depth (Fig. 4a). The heterogeneity in diffusion coefficients was manifested as an increased standard deviation associated with the estimates of diffusion coefficient (Fig. 4a).

At deeper sections of the microcolony (25 μm shown in Fig. 4c and 20–30 μm shown in Supplementary Fig. 13), the fluorescent image had three distinct regions, a dim outside, a bright peripheral interior and a dim central interior region. This shows that even though the positively charged molecule had a higher concentration within the biofilm when compared to the exterior, its distribution is not uniform throughout the biofilm.

Similar to the fluorescence intensity, the diffusion maps (25 μm shown in Fig. 4d and 20–30 μm shown in Supplementary Fig. 13) displayed three regions, a heterogeneous exterior, homogeneous peripheral interior and a heterogeneous central interior. A comparison of Fig. 4c, d shows that bright fluorescent regions (marked I-B in Fig. 4c) correspond to the homogeneous regions based on diffusion coefficient and dim fluorescent regions (marked I-D in Fig. 4c) exhibited greater heterogeneity in their diffusion profiles.

The fraction of the slow moving particles in the deep interior of the biofilm increased beyond 20 µm (Fig. 4a, inset). The fraction of slow particles was the lowest in the periphery of the biofilm at 25 µm (Fig. 4e). The slowly diffusing particles may result from strong interactions with the EPS leading to an increased fraction of slow moving particles in the central interior region of the biofilm.

### SPIM-FCS measurements of diffusion of labelled antibiotics

**Fig. 5:**
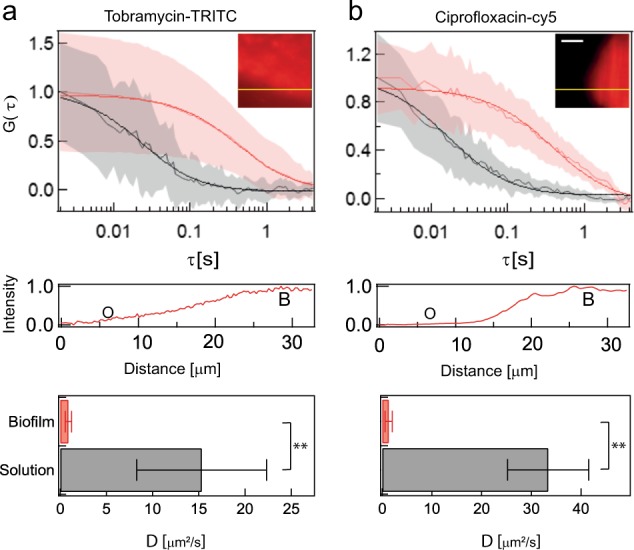
SPIM-FCS measurements of diffusion of labelled antibiotics: The average autocorrelation curve of fluorescently labelled tobramycin and ciprofloxacin is shown in red in **a** and **b**, respectively. The light red shading around the curve is the error over an average of 100 pixels. The curve in black in **a** and **b** is the average autocorrelation curve inside the biofilm. The inset shows a fluorescence image of a biofilm to which fluorescently labelled tobramycin and ciprofloxacin have been added. The intensity profile along the yellow line in the insets is shown below the autocorrelation plots. **b** denotes the biofilm. Bar charts show the diffusion coefficients of antibiotics within the biofilm and in solution. The measurements in solutions are an average of three measurements each. In the case of biofilms, these are an average of 11 and 8 microcolonies for tobramycin (*p* = 0.007) and ciprofloxacin (*p* = 0.006), respectively. In the autocorrelation plots, the raw data are shown in grey and light red while the fitted data are shown in black and red. The significance levels were determined using a two-sided Wilcoxon rank test. The error bars in **a** and **b** show the standard deviation. The scale bar in the inset of **b** measures 10 µm

Given that DEAE-dextran preferentially partitioned into the biofilm, we then tested whether a positively charged aminoglycoside antibiotic tobramycin,^41^ exhibited the same behaviour. Similar to DEAE-dextran, positively charged, TRITC-labelled tobramycin penetrated into the biofilms and preferentially partitioned into the biomass (Fig. 5a, inset).

The intensity profile across the biofilm border clearly shows increased intensity inside the biofilm compared to the exterior (Fig. 5). There was also a 15-fold statistically significant reduction (*N* = 11; *p* = 0.007) in the diffusion coefficient of fluorescently labelled tobramycin from solution (15.3 ± 7.0 µm^2^ s^−1^) to the interior of the biofilm (0.9 ± 0.3 µm^2^ s^−1^) (Fig. 5a). Similar to tobramycin, a neutral fluoroquinolone ciprofloxacin,^42^ fluorescently labelled with Cy5, also accumulated in the biomass and exhibited a 26-fold statistically significant reduction (*N* = 8; *p* = 0.006) in diffusion coefficient from solution (33.4 ± 8.1 µm^2^ s^−1^) to the biofilm interior (1.3 ± 0.7 µm^2^ s^−1^) (Fig. 5b).

### The effect of dispersal of *P. aeruginosa* biofilms on molecular diffusion

Next, we probed changes in diffusion coefficient of 2 MDa TRITC-dextran for approximately 8 h as the microcolony was undergoing induced dispersal. The intracellular level of c-di-GMP regulates the transition from biofilm to planktonic phenotype. The cellular concentration of bis-(3′–5′)-cyclic dimeric guanosine monophosphate (c-di-GMP) is dynamically controlled by the opposing activities of multiple diguanylate cyclases (formation of c-di-GMP) and phosphodiesterases (degradation of c-di-GMP).

**Fig. 6:**
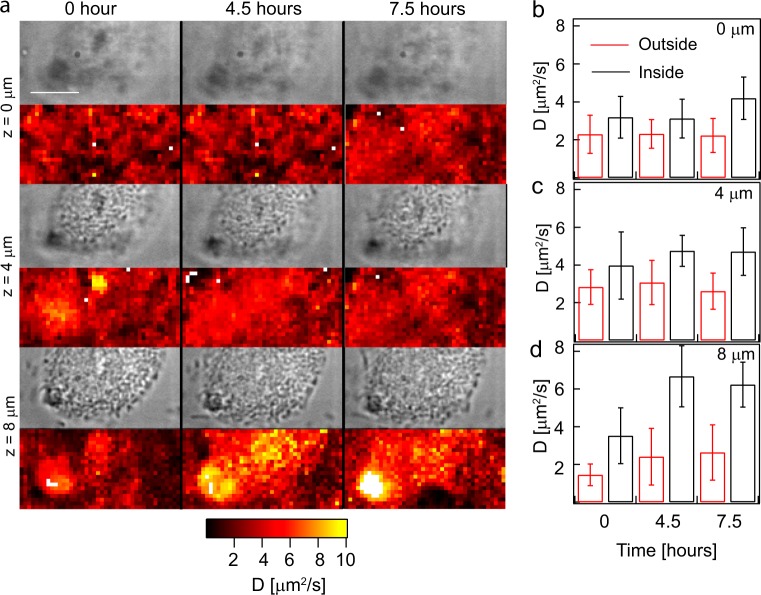
The effect of dispersal of *P. aeruginosa* biofilms on molecular diffusion: **a** is a montage of the *z* stacks and time-lapse of the wide field and parametric diffusion maps at various times post induction using 1% arabinose of *P. aeruginosa* PA01 eGFP pBAD yhjH biofilm grown in ABGC gentamycin media. The diffusion coefficients of 2 MDa dextran measured at different depths and at different time points were quantified and shown in **b**–**d**. The error bars in **b**–**d** show the standard deviation. The scale bar in **a** measures 10 µm

In the *P. aeruginosa* pBAD *yhjH* strain, a phosphodiesterase gene was cloned with an arabinose inducible promoter^43,44^ to induce biofilm dispersal. The microcolony was imaged for 8 h after the addition of the inducing molecule-arabinose. The diffusion coefficient inside the microcolony 7.5 h post induction increased twofold when compared to that in the solution at *z* = 8 µm (Fig. 6d). The periphery between the exterior of the microcolony and the interior of the microcolony is discernible in the diffusion maps at the bottom most plane after at least 4 h of induction (Fig. 6). A similar trend was also seen in another microcolony (Supplementary Fig. 14) in which the diffusion coefficient inside the microcolony was higher than that of the outside at 7.5 h post induction in the bottom most plane at *z* = 15 µm. However, only 2 out of 10 colonies imaged exhibited this behaviour within 8 h. The other eight colonies did not show an increase in diffusion coefficient inside the microcolony post induction. This suggests that SPIM-FCS is able to monitor changes in physicochemical properties of the biofilm during dispersal before the detection of biomass loss.

## Discussion

In this study, we used SPIM-FCS to measure 3D diffusion profiles of molecules of different size and charge in biofilms of various sizes, providing insights into biofilm structure from attachment to dispersal. SPIM-FCS is an ideal tool for this task, due to the reduced photobleaching and photodamage and increased speed of measurements compared to confocal FCS. Performing diffusion studies at the single microcolony level allowed quantification and visualization of the heterogeneity of diffusion and structure in biofilms.

The EPS matrix of biofilms acts as a molecular sieve^3^ and restricts the penetration of molecules into the biofilm larger than its pore size. Previous estimates of pore size ranged from 500 to 1000^45^ and 125 nm^27,46^ by different methods for *P. aeruginosa* PAO1, the same strain used here. In the case of *Pseudomonas fluorescens*, loose flocs had an effective pore size of 50 nm, which decreased to 10 nm for dense biofilms.^24^ In this study, molecules up to hydrodynamic radius of 27 nm penetrate into the biofilm, setting a lower limit for the pore size and diffuse slower in biofilms when compared to free solution. Even though there is variability in the beginning of microcolony formation, a reduction in porosity occurs as they increase in size. Despite the fact that 2 MDa TRITC-dextran penetrated into the biofilm, there was a concentration difference between the outside and the inside of the biofilms. The reduction in diffusion coefficient of probes exogenously added to biofilms is best described by the obstruction model of diffusion in hydrogels.^47,48^ In this model, the biofilm acts as a sieve and solutes with sizes smaller than the pore size of the biofilm can diffuse into the biofilm. The mobility inside the biofilm is directly related to the void fraction of the biofilm and inversely related to the tortuosity of the biofilm. This may be because as the size of the molecule increases, there is hindered diffusion^49^ or an entanglement with the polymeric material that constitutes the EPS.^50,51^

We parametrized the geometry of a 3D microcolony as two independent parameters, lateral diameter and axial depth. While there was no statistically significant difference in diffusion coefficient across days post inoculation, there was a correlation between the diffusion coefficient and the diameter of the microcolony in which it was measured. Small colonies had a higher average diffusion coefficient than larger colonies. The heterogeneity in diffusion coefficients of colonies with a diameter of 20–30 µm suggests that the crosslinking of polymers in the small colonies may differ sufficiently to alter diffusion of these molecules. This suggests that the matrix composition of the microcolonies may change over time leading to changes in crosslinking and porosity. This is also consistent with AFM data, showing that the Young’s modulus of early stage biofilms is less than that of older, larger microcolonies.^52^ The correlation between diffusion coefficient and size of a microcolony also suggests that microcolony size is a more robust metric than age when comparing and characterizing biofilms.

While colony size, rather than age, was a better predictor of diffusion properties, colonies of the same size can have very different 3D structures and this may also influence diffusion behaviours. In a subset of colonies (~20%) we found clear changes with depth, manifested as decreased signal-to-noise ratios, larger COVs and broader distributions for the measured diffusion coefficients. Our current explanation is that as the depth increases, the crosslinking of the polymers in the EPS increases, leading to a decrease in porosity and a more heterogeneous environment, thus leading to reduced penetration of the probe to deeper regions of the biofilm. In the other 80% of microcolonies, the probes penetrated evenly throughout the colony. This suggests that in a given population of microcolonies, there is variability in the crosslinking of EPS with depth of the microcolony, independent of the diameter.

The self-produced matrix of EPS in *P. aeruginosa* consists of positively charged (polysaccharide-Pel,^53^) negatively charged (eDNA,^54^ alginate,^55^ filamentous phage Pf4^56^ and neutral molecules (polysaccharide-Psl.^54^) The heterogeneity in the distribution of the 150 kDa FITC-DEAE-dextran in different regions of the biofilm is an indication of a non-uniform charge distribution which might arise due to the variation in the spatial distribution of charged molecules within a biofilm.^57^

We hypothesize that the decreased mobility of the positively charged DEAE-dextran molecules might be due to either replacement of cationic molecules in EPS or electrostatic interactions with negatively charged molecules in the EPS. Positively charged exopolysaccharide-Pel is a cross-linker found in the EPS. The decreased mobility of the DEAE-dextran molecule might be due to the replacement of Pel in the EPS network by DEAE-dextran. Apart from replacement, the reduction in mobility of positively charged molecules might also be due to interactions with the negatively charged components of EPS matrix such as filamentous phage Pf4 or eDNA. Cationic molecules bind and cross-link Pf4 phages^56^ and such binding will lead to a reduction in mobility. eDNA chelates cations as they pass into the biofilm matrix^59^ through electrostatic interactions.^58^

Similar to observations in this study, using SPT, it was shown that positively charged molecules accumulate within the biomass.^27^ In contrast to *P. aeruginosa*, cationic molecules did not penetrate biofilms of *Lactococcus lactis* or *Stenotrophomonas maltophilia*^19^ and this may be a consequence of the biofilm matrices being composed of different biopolymers for the various bacteria.

To monitor bacteria in different physiological states during the biofilm life-cycle, we performed SPIM-FCS on a strain undergoing induced dispersal. After inducing dispersal, there was an increase in the diffusion coefficient inside the biofilm that was even higher than diffusion as measured in solution. We hypothesize that this may be due to the fluidic activity during the seeding dispersal^59^ and would be consistent with a breakdown of the matrix and increased motility of the bacteria, which is required for bacteria to escape the matrix during dispersal.

Eighty per cent of the colonies did not show an increase in diffusion coefficient. We speculate that the dispersal process was not induced in these colonies. The low efficacy of dispersal could be due to the use of arabinose induction media containing glucose. Glucose is not completely exhausted in the medium during the induction and hence the cells are not physiologically prepared for dispersal. On the contrary, the cells experiencing starvation are more prone to dispersal due to changes in c-di-GMP. Determination of the timeframe in which dispersal is induced will be useful for the fields of clinical microbiology, internal medicine and pharmacology as therapeutic solutions have advocated the use of dispersal agents in addition to antibiotic treatment^60^ along with time-dependent dosage of antimicrobials.^61^

Similar to observations by Walters et al.,^33^ both ciprofloxacin and tobramycin penetrated into *P. aeruginosa* biofilms. Ciprofloxacin and tobramycin accumulated in the *P. aeruginosa* biofilm and exhibited reduced diffusion compared to solution. The reduction of the diffusion coefficient of tobramycin is in agreement with previous observations of reduced tobramycin diffusion by a disc diffusion assay in the presence of alginate^62^ and can also be explained by the interactions with the EPS of *P. aeruginosa.*^63^ The estimates of the increase in concentration and decrease in diffusivity inside the biofilm for tobramycin is useful when modelling the bacterial biofilm as a reaction-diffusion system.^64^ The increase in concentration of tobramycin inside seaweed alginate beads was earlier explained by theoretical studies using a reaction-diffusion model with non-specific power law binding to polyanion matrix components.^65^ Our measurements showed a 1.5-fold increase in intensity of labelled tobramycin inside the biofilm when compared to the exterior.

Results from Landry et al.^66^ suggest that the efficiency of penetration of tobramycin into “flat, homogeneous” biofilms is higher than penetration into biofilms characterized with “large cell aggregates”. In the case of “fully matured biofilms with large cell aggregates”, tobramycin was sequestered in the periphery of the microcolonies.^67^ We observed penetration of tobramycin into the biofilm microcolony and did not see sequestration of tobramycin in the periphery since the penetration experiments were performed on flat biofilms, which did not have mushroom shaped structures, but rather, the microcolonies appear as flattened mounds of cells.

Previous reports and our own results have shown that the biofilm does not represent a diffusion barrier for antibiotics^26,28^ in *P. aeruginosa* biofilms despite a reduction in diffusion coefficient. Together, our results suggest that diffusion limitations do not contribute to the observed tolerance in clinically relevant biofilms for antimicrobials. This suggests that other mechanisms that convey antibiotic tolerance in biofilms,^64,68^ such as physiological differentiation, play a more important role in biofilm tolerance to antibiotics than the extracellular matrix.

It is well known that biofilms exhibit physiological, architectural and biochemical heterogeneity. Our results suggest that there is also considerable heterogeneity in the diffusion of molecules in biofilms. It remains to be tested whether the physiological heterogeneity in biofilms is a cause or an effect of heterogeneity in diffusion. The mobility of molecules diffusing inside biofilms obtained in this study is not only useful in gaining an understanding of the fundamental principles governing diffusion in a heterogeneous biofilm system but also has utilization in the pharmacokinetic modelling of drugs and in mass transfer modelling in bioreactors for wastewater treatment. Information about the effects of molecular properties on their diffusion in biofilms along with the effects of biofilm physiology on molecular diffusion is invaluable in the selection and design of agents that can effectively penetrate the biofilm matrix to reach the target site of action. For example, the results from this study could be used to design improved drug delivery systems. Based on the signal-to-noise ratio of the raw data of the autocorrelation curves and imaging studies using the three charged molecules, we suggest that a carrier with an outer, positively charged surface would have the highest penetration compared to neutral or negatively charged molecules. Quantification of the diffusion coefficient of molecules along with their variation in living biofilms will aid in efficient strategizing of the kinetics upon the addition of biofilm removal agents in clinical and industrial settings.

## Methods

*P. aeruginosa* PA01 pBAD *yhjH*,^43^ PA01 wild type, PA01 eGFP and PA01 pBAD *yhjH* eGFP have been used in this study. Single colonies were inoculated into 10 mL Luria-Bertani (LB) medium (10 g L^−1^ tryptone, 5 g L^−1^ yeast extract, 10 g L^−1^ sodium chloride) and incubated overnight at 25 °C to prepare the inoculum. For plasmid maintenance in *P. aeruginosa* PA01 pBAD *yhjH*, LB was supplemented with 30 µg mL^−1^ gentamycin.

TRITC-dextrans of various molecular weights (2 MDa, 500, 150, 70, 40, 20 and 4 kDa), diethyl aminoethyl, carboxy methyl dextran and neutral 150 kDa FITC-dextran were obtained from TdB Consultancy (Uppsala, Sweden), and dissolved in water to prepare stock solutions. TRITC-labelled tobramycin and cy5-labelled ciprofloxacin were purchased from Bioconjugate Technology Company (Scottsdale, AZ, USA). Except for the 2 MDa conjugates, SPIM-FCS experiments on alginate beads were done with 100 nM TRITC-dextran. In the case of FITC-dextran, 250 nM and 1 µM were used for alginate beads and biofilms. In the case of 2 MDa, 10 nM was used for alginate beads and biofilms for confocal and SPIM-FCS. In the case of confocal FCS, 30–250 nM was used (4, 20, 40, 70, 150 and 500 kDa). A concentration of 100 nM of labelled tobramycin and ciprofloxacin was used for the experiments.

### Generation of PA01 eGFP pBAD *yhjH* strain

The eGFP-expressing strain carries a gentamycin resistance cassette as part of the transposon used to introduce the *gfp* gene. Therefore, this gentamycin cassette was first removed from the chromosome using the FLIP recombinase system and selected for gentamycin sensitivity. Subsequently, a gentamycin-sensitive isolate was transformed with the pBAD yhjH plasmid and selected on gentamycin plates.

### Biofilm growth conditions

*P. aeruginosa* PA01 eGFP pBAD yhjH was grown in AB^69^ minimal medium containing 15.1 mM (NH_4_)_2_SO_4_, 33.7 mM Na_2_HPO_4_, 22 mM KH_2_PO_4_, 0.05 mM NaCl, 1 mM MgCl_2_, 100 µM CaCl_2_ and 1 µM FeCl_3_. The medium was supplemented with 0.2% glucose (G) and 0.2% casamino acids (C). This medium is referred to as ABGC medium. For dispersal experiments, for pBAD yhjH plasmid maintenance, 30 µg mL^−1^ gentamycin was added to the medium (ABGC gen media). Media was pumped at a rate of 5 mL h^−1^ using a peristaltic pump (Cole Parmer Masterflex (Vernon Hills, IL, USA)—flow speed at 8) into the flow cell through fluorinated ethylene propylene (FEP) tubing.

A single colony was inoculated into 10 mL medium and incubated at 25 °C and 200 rpm overnight. In the case of PA01 wild type (used in all experiments except those described in Fig. 6 and Supplementary Fig. 14), the overnight culture was diluted to an optical density (OD_600_) of 0.3 before injection into flow setup. Flow was stopped before inoculation and resumed 1 h after inoculation. In the case of PA01 eGFP pBAD yhjH, the overnight culture was subcultured till the OD_600_ reached 0.1 and then injected as inoculum into the flow chamber. The OD_600_ was chosen to be 0.1 since live wide-field microscopy showed a reduction in biomass visually after arabinose induction.

### SPIM-FCS

Biofilms were grown in 3 cm long square profile FEP^70^ tubes (nominal size 2 mm × 2 mm; Adtech, UK) in minimal medium. FEP was chosen because the refractive index of the material matches that of the water used as immersion medium for the objective and because the tube was sturdy enough to withhold shear stresses due to the flow in the flow chamber. The biofilms grew on all four sides of the tube. The biofilms growing on the side closest to the detection objective were used for imaging and spectroscopy. The position of light sheet is fixed in the microscope, hence different regions of the biofilm can be illuminated by moving the sample relative to the light sheet. Data from different microcolonies from different flow experiments were compiled to estimate diffusion coefficients.

FEP tubes were removed from the flow cells and one end was sealed with glue. Colonies were imaged using Carl Zeiss commercial light sheet microscope Z.1 (referred to as light sheet 1). Two different EMCCD cameras (Andor, Belfast, UK) controlled using Andor Solis software were used as the detectors (Andor 860-referred to as camera 1 and Andor 897, referred to as camera 2). Measurements for Fig. 1 and Supplementary Fig. 8 were performed in camera 2 while the rest of experiments involving biofilms in this manuscript were performed with camera 1. Unless otherwise stated in the figure legend, Andor iXon 860 was used for the detection.

A laser power of 1–3 mW was used for illumination. LSFM×10/NA0.2 served as the illumination objective while a WPlan-Apochromat ×63/NA1.0 was used as the detection objective. After blocking the excitation light, the detected fluorescence was filtered using SBSLP510, LP565 and 660DF50 filters for 488, 561 and 638 nm lasers, respectively. Laser blanking was switched off and a macro was used to create a static light sheet.

The metadata showed that the scaling X and Y were 72 nm. This corresponded to ×90 magnification. A frame length of 50,000 with 0.002 s exposure time was used throughout the paper except for the data in Fig. 1. A frame length of 100,000 frames with 0.004 s exposure time was used for the experiments in Fig. 1. Data analysis was performed using an ImageJ plugin called Imaging FCS 1.47 (ref. ^71^). The fitting model used to determine the diffusion coefficient is provided in the manual of the software. Polynomial order 4 bleach correction was used. The data were binned 4 × 4 in the case of Supplementary Fig. 1. The rest of the data is binned 3 × 3. The fitting model used was

1 $$G\left( \tau \right) = \frac{1}{N}\frac{{g\left( \tau \right)}}{{g\left( 0 \right)}},$$

where

$$\begin{array}{l}g\left( \tau \right) = \left( {\frac{{\sqrt {4D\tau + \omega _{xy}^2} }}{{a\sqrt \pi }}\left( {{\mathrm e}^{ - \frac{{a^2}}{{4D\tau + \omega _{xy}^2}}} - 1} \right) + {\mathrm {Erf}}\left( {\frac{a}{{\sqrt {4D\tau + \omega _{xy}^2} }}} \right)} \right)^2\\ \times \left( {1 + \frac{{4D\tau }}{{\omega _z^2}}} \right)^{ - \frac{1}{2}} \;+\, G_\infty,\end{array}$$

where *a* is the pixel size, *τ* is the lag time, *N* is the number of particles, *D* is the diffusion coefficient, *ω*_*xy*_ is the PSF in the *xy* direction while *ω*_*z*_ is the thickness of the light sheet. The procedure to determine *ω*_*xy*_ and *ω*_*z*_ is described in Supplementary Methods. The experimental values are also tabulated in the same section. In the case of two components, the fitting model is given by

2 $$G\left( \tau \right) = \frac{{\left( {1 - F_2} \right)\frac{{g_1\left( \tau \right)}}{{g_1\left( 0 \right)}} + F_2\frac{{g_2\left( \tau \right)}}{{g_2\left( 0 \right)}}}}{N}\, + G_\infty,$$

where *F*_*2*_ is the fraction of the slow moving particle obtained from curve fitting. *D*_*1*_ and *D*_*2*_ are the diffusion coefficients of the fast and the slow moving particles respectively.

### Zeta potential measurements

The zeta potential of untreated, DEAE and CM dextran were measured using Malvern Zetasizer Nano (Worcestershire, UK). A concentration of 10 mg mL^−1^ was used for all the three probes. The probes were dissolved in DI water for the measurement.

### Statistical tests

All the statistical tests were performed in Igor Pro^©^ (Wavemetrics, OR, USA).

### Reporting summary

Further information on research design is available in the Nature Research Reporting Summary linked to this article.

## Supplementary information

Supplementary material

Reporting summary

## Data Availability

The datasets generated during the current study are available from the corresponding author on reasonable request.
