# Supplementary material for: Single microcolony diffusion analysis in Pseudomonas aeruginosa biofilms
Source: NPJ Biofilms Microbiomes. 2019 Nov 8;5:35. doi: 10.1038/s41522-019-0107-4 (PMC6841743; doi:10.1038/s41522-019-0107-4)
Supplement: Supplementary file 1 — Supplementary material [file 41522_2019_107_MOESM1_ESM.pdf]

**Supplementary material for**

# Single microcolony diffusion analysis in *Pseudomonas aeruginosa* biofilms

Jagadish Sankaran[1, 2], Nicholas John Tan Jie Hao[3, 4], But Ka Pui[2, 5], Yehuda Cohen[3,4], Scott. A. Rice[3,4,6,*] and Thorsten Wohland[1,2,5,*]

[1]*Departments of Biological Sciences, National University of Singapore, 117558, Singapore*

[2]*Centre for BioImaging Sciences, National University of Singapore, 117557, Singapore*

[3]*Singapore Centre for Environmental Life Sciences Engineering and* [4]*School of Biological Sciences, Nanyang Technological University, 637551, Singapore*

[5]*Department of Chemistry, National University of Singapore, 117543, Singapore*

[6]*ithree Institute, University of Technology Sydney, Sydney, Australia*

**mail to: rscott@ntu.edu.sg; mail to: twohland@nus.edu.sg*

**Supplementary note 1: Accuracy and precision of diffusion coefficients obtained from Imaging FCS**

The diffusion coefficient of TRITC labelled dextrans with molecular weights ranging from 4 kDa to 2 MDa were estimated using SPIM-FCS in order to characterize the reliability of estimates from this commercially available instrument. The figure of merits for the accuracy and precision of estimates of diffusion coefficient from SPIM-FCS are the root mean square average percentage relative error and coefficient of variation (COV) respectively.

$$\%\ Relative\ Error = \frac{D_{Experimental} - D_{Theoretical}}{D_{Theoretical}} * 100$$ Supplementary Eq. 1

$$Coefficient\ of\ variation = \frac{Standard\ deviation\ of\ the\ distribution}{Mean\ of\ the\ distribution}$$ Supplementary Eq. 2

The root mean square average of the % relative error is presented in Table S1.

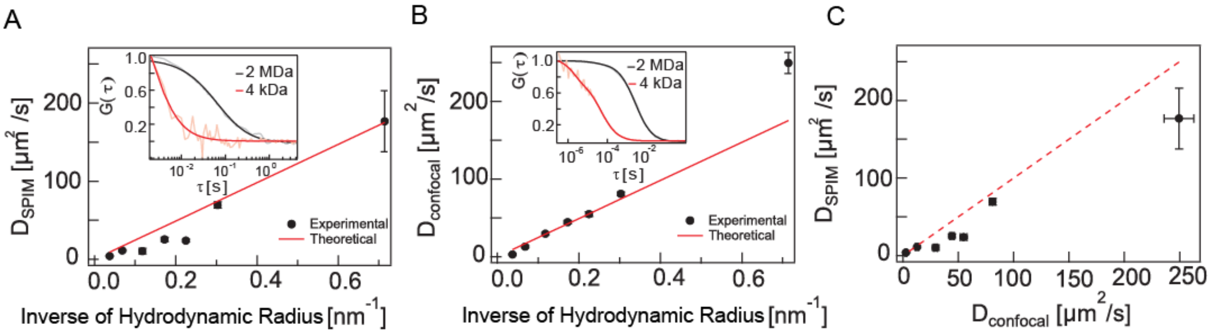


***Supplementary Figure 1**: A and B are plots of diffusion coefficients obtained for TRITC-dextrans of different molecular weights in SPIM-FCS and Confocal-FCS respectively. The insets represent autocorrelation curves for 2 MDa and 4 kDa molecules. The 2 MDa curve is right shifted when compared to 4 kDa. The insets also highlight that the x-axis in Imaging FCS and confocal FCS cover different timescales. The raw data is shown in light red and grey while the fitted data is shown in black and red in the inset. C is a scatter plot of values obtained in confocal FCS against those obtained from SPIM-FCS. The dotted red line is a line drawn at 45° for visual aid.*

**Accuracy and precision of estimates from SPIM-FCS**

The root mean square average of the % relative error was 43% for the light sheet, while it was 32% for the confocal FCS. The diffusion coefficients obtained from SPIM-FCS and Confocal FCS are shown in Supplementary Tables 1, 2 and Supplementary Fig. 1. Both techniques show deviations from the theoretical curve, which we attribute to the size distribution. Two out of seven dextrans, had < 25% absolute error in diffusion coefficients in SPIM-FCS whereas 5 out of 7 values had < 25% absolute error in confocal FCS (Supplementary Table 1).

Supplementary Fig. 1C is a scatter plot between the diffusion coefficients obtained from SPIM-FCS and confocal FCS. The dotted red line is a line drawn at 45° for visual aid. The majority of the values are below the 45° line, which indicates that values from SPIM-FCS are less than those obtained from confocal FCS. Except for the 2 MDa TRITC-dextran, the diffusion coefficient obtained from SPIM-FCS was less than that of confocal FCS (Supplementary Table

1). We currently attribute this to the limited time resolution of the camera used for detection. The limited time resolution of the camera can be overcome by the use of cameras that can measure at 10000 fps. At an exposure time of 2 ms, the first point in the autocorrelation curve is calculated at a lagtime of 2 ms. The plateau of the autocorrelation function is not measured in the case of fast moving molecules (4 kDa here) if measured using a 2 ms exposure time since the autocorrelation curve has decayed considerably when compared to the autocorrelation curve of 2 MDa. Autocorrelation curves for the 2 MDa and 4 kDa TRITC-dextrans are shown in the insets of Supplementary Fig. 1A and B. Supplementary Fig. 1B inset shows that in the case of confocal FCS, the plateau was captured for both the 2 MDa and 4 kDa TRITC-dextrans. However, the plateau was not observed in the case of SPIM-FCS for 4 kDa as seen by the autocorrelation function in the inset.

**Supplementary Table 1. Diffusion coefficients from SPIM-FCS and confocal FCS**

| **M.W** | $R^{+}$ **[nm]** | $D_T^{*}$ [$\mu m^2 s^{-1}$] | $D_{SPIM\text{-}FCS}$ [$\mu m^2 s^{-1}$] | **%Error$_{SPIM\text{-}FCS}$** | **D$_{Confocal\ FCS}$** [$\mu m^2 s^{-1}$] | **%Error $_{Confocal\ FCS}$** |
|---|---|---|---|---|---|---|
| **2 MDa** | 27 | 9.1 | 4.1 ± 0.6 | -54.9 | 2.8 ± 0.8 | -69.2 |
| **500 kDa** | 12.7 | 16.7 | 11.4 ± 1.1 | -31.7 | 12.8 ± 1.5 | -23.3 |
| **150 kDa** | 8.5 | 28.9 | 10.5 ± 3.5 | -63.7 | 29.6 ± 1.8 | 2.6 |
| **70 kDa** | 6 | 42.3 | 25.4 ± 2.7 | -39.9 | 44.3 ± 2.1 | 4.8 |
| **40 kDa** | 4.45 | 55.1 | 24.0 ± 1.6 | -56.5 | 54.8 ± 3.1 | -0.6 |
| **20 kDa** | 3.3 | 74.3 | 69.6 ± 4.1 | -6.4 | 81.0 ± 2.4 | 9.0 |
| **4 kDa** | 1.4 | 175.1 | 176.8 ± 39.2 | 0.9 | 249.5 ± 13.4 | 42.4 |
| **RMS average** | | | | 43 | | 32 |

$^{+}R$ is the hydrodynamic radius, $^{*}$ $D_T$ is the theoretical value of diffusion coefficient.

**Supplementary Table 2. Precision of estimates from SPIM-FCS and confocal FCS**

| **M.W** | ***R* [nm]** | **% COV1 for SPIM-FCS** | **% COV2 for SPIM-FCS** | **% COV for confocal FCS** |
|---|---|---|---|---|
| **2 MDa** | 27 | 14 | 34.4 ± 12.6 | 28.5 |
| **500 kDa** | 12.7 | 10.0 | 19.5 ± 4.6 | 11.7 |
| **150 kDa** | 8.5 | 33.4 | 35.7 ± 15.2 | 6.0 |
| **70 kDa** | 6 | 10.6 | 17.9 ± 4.3 | 4.7 |
| **40 kDa** | 4.45 | 6.6 | 21.9 ± 6.4 | 5.6 |
| **20 kDa** | 3.3 | 5.8 | 13.2 ± 1.7 | 2.9 |
| **4 kDa** | 1.4 | 22.1 | 35.6 ± 14.2 | 5.3 |
| **Mean** ± SD | | 14.6 ± 9.9 | 25.4 ± 9.5 | 9.3 ± 8.9 |

$^{+}R$ is the hydrodynamic radius

COV1 for SPIM-FCS (Supplementary Table 2) is the ratio of the standard deviation to the mean of the values provided in $D_{SPIM\text{-}FCS}$. For every molecular weight, a series of measurements in SPIM-FCS are performed. Each measurement has a mean and a standard deviation. Every mean and standard deviation can be transformed into COV. The average and standard deviation from this distribution of COV is shown in COV2. Both COV1 and COV2 are higher than the coefficient of variation of confocal FCS, which indicates that confocal FCS yields more precise estimates than SPIM-FCS. Both COV1 and COV2 are less than 30% for SPIM-FCS. The root mean square average of the % relative error of SPIM-FCS is 11% more than that of confocal

FCS. This suggests that SPIM-FCS is capable of measuring diffusion coefficients with ~40% error in accuracy and ~25% precision of the estimated value.

## Supplementary note 2. SPIM-FCS measurements on biofilm mimics to characterize the ability of the technique to probe inhomogeneous environments

We performed Imaging FCS studies on biofilm mimics to ascertain the ability of the technique to distinguish inhomogeneous environments. In order to mimic biofilms microcolonies, we used alginate beads since alginate is known to be a constituent of the polysaccharide matrix. This set of measurements were performed on a home built SPIM-FCS set up which has already been validated and used for SPIM-FCS measurements on mammalian cells so far[1-3]. Probes of different sizes and charges were added to alginate mimics to understand the role played by size and charge in determining the penetration and diffusion of molecules. The results obtained from Imaging FCS were compared with that of a well-established technique-confocal FCS (Supplementary notes 3).

### SPIM-FCS experiments to probe diffusion of neutral molecules of different sizes on biofilm mimics

TRITC labelled 20 kDa, 150 kDa and 2 MDa dextrans were added to alginate beads. There was a reduction in fluorescence intensity inside the bead when compared to the exterior of the bead (Supplementary Fig. 2A-C). The diffusion maps (Supplementary Fig. 2 D-F) show that the bright regions corresponding to regions of high mobility are outside of the bead for the 20 and 150 kDa molecules. The average diffusion coefficient in the exterior was 55.3 ± 2.3, 14.7 ± 2.5, 1.3 ± 0.4 $\mu m^2 s^{-1}$ for 20 kDa, 150 kDa and 2 MDa respectively. Inside the bead, the average diffusion coefficient was 14.5 ± 3.4, 1.4 ± 1.1 and 1.6 ± 1.1 $\mu m^2 s^{-1}$ respectively. The 20 kDa dextran exhibited less reduction in diffusion coefficient after penetrating into the bead when compared to the 150 kDa as seen by the ratio of diffusion coefficients, 3.6 and 7.3 for 20 and 150 kDa, respectively, between the exterior and the interior (Supplementary Table 3).

The diffusion maps at four different positions of the bead (D-F) were quantified to determine heterogeneity of the environment within the beads. In the case of 20 kDa and 150 kDa, all of the diffusion maps show that the diffusion coefficients inside the beads are smaller than outside. However, in the case of 2 MDa, there is large variability of diffusion coefficients in the interior, suggesting a heterogeneous structure within the bead. We quantified the heterogeneity as the global coefficient of variation for the diffusion coefficient ratio of the exterior to the interior, which is 30% for 20 kDa while more than 100% for 2 MDa (as seen in Supplementary Table 3).

The autocorrelation functions for 20 kDa and 2 MDa in the exterior and interior of the alginate bead are shown in Supplementary Fig. 2G and H. The 20 kDa molecule diffuses slowly in the interior of the bead when compared to the exterior as seen in in Supplementary Fig. 2G. Apart from the change in rate of decay of the autocorrelation function, it is observed for both molecules that there is a reduction in amplitude in the interior of the bead when compared to the exterior. There is a 60% drop in amplitude for the autocorrelation function for the 20 kDa molecule while there is a 75% drop in amplitude for the autocorrelation function for the 2 MDa molecule. This is a consequence of the lower signal-to-noise ratio within the beads (Ref. Supplementary notes 4).

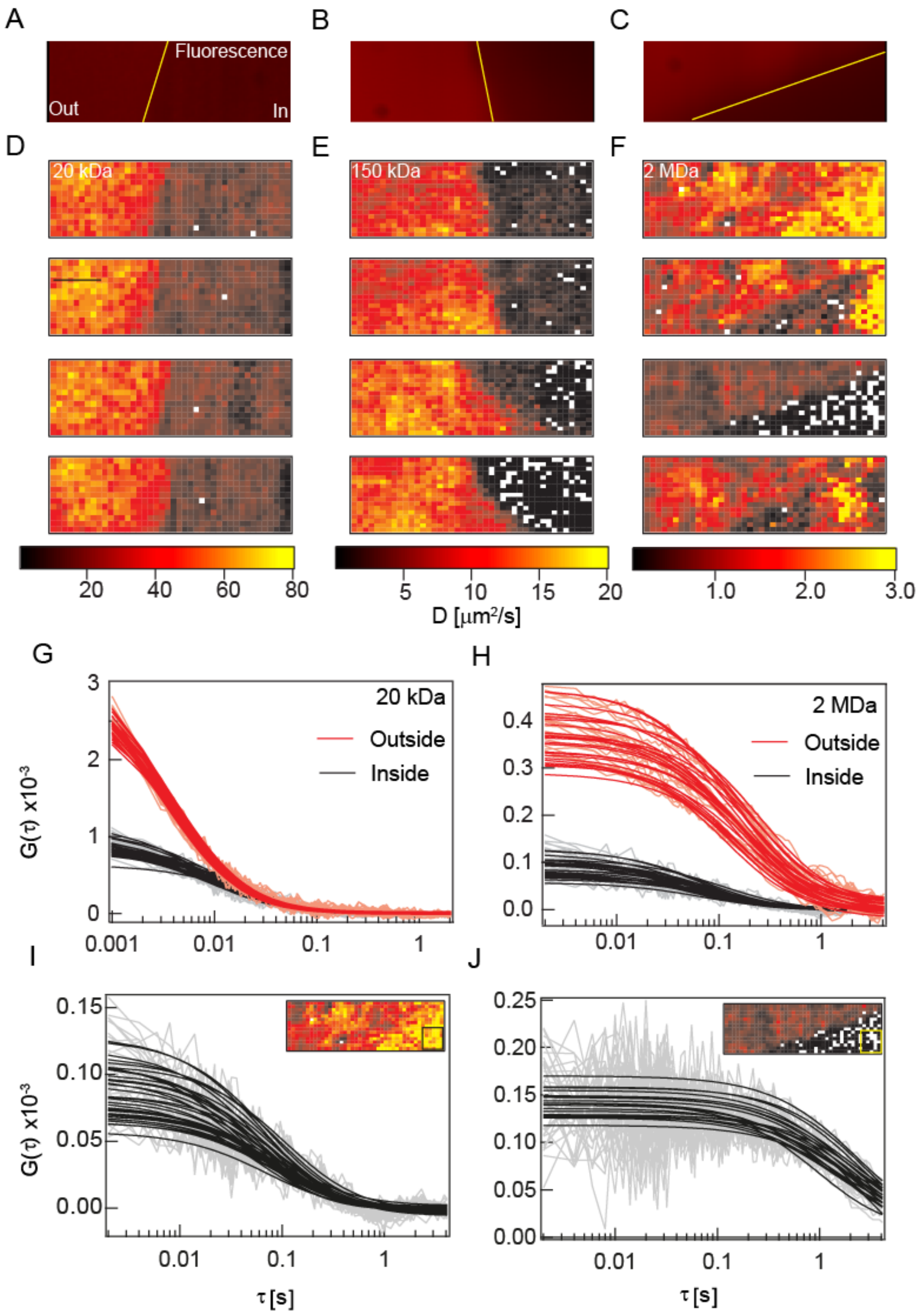


***Supplementary Figure 2**: Representative fluorescent images of beads obtained using the light sheet microscope are shown in A, B and C for 20 kDa TRITC-dextan, 150 kDa TRITC-dextran and 2 MDa TRITC-dextran respectively. In all the three cases, the alginate bead is in the right side of the image. Diffusion maps obtained from different positions of an alginate bead (20 kDa: D, 150 kDa: E and 2 MDa: F). It is to be noted that the ranges of diffusion coefficient covered in diffusion maps shown in D, E and F are different. G and H show autocorrelation curves in the exterior (red) and interior (black) of the bead for 20 kDa TRITC-dextran and 2 MDa TRITC-dextran respectively. The raw data is shown in light red and grey in the exterior and interior respectively. I and J show autocorrelation curves from the interior of the alginate bead where 2 MDa-TRITC-dextran was added. I and J correspond to the boxed region in the first and the third position shown in F (also shown in the insets). The scale bar shown in D is 10 μm.*

**Supplementary Table 3. Diffusion coefficients of TRITC labelled dextrans in alginate beads obtained from SPIM-FCS**

| **Size** | ***D*** **Exterior** **[$\mu m^2 s^{-1}$]** | ***D*** **Interior** **[$\mu m^2 s^{-1}$]** | **Ratio*** | **N[@]** | **Ratio** | **% Global COV for Ratio** | **$N_{pos}$** | **N[&]** |
|---|---|---|---|---|---|---|---|---|
| | Sample data** | | | | Population data[+] | | | |
| **20 kDa** | 52.9 ± 7.9 | 14.8 ± 5.2 | 3.6 ± 1.4 | 100 | 4.0 ± 1.2 | 30 | 6 | 3444 |
| **150 kDa** | 10.3 ± 2.3 | 1.4 ± 1.0 | 7.3 ± 5.5 | 100 | 16.6 ± 16.1 | 96.9 | 5 | 4284 |
| **2 MDa** | 1.5 ± 0.4 | 1.9 ± 1.1 | 0.8 ± 0.5 | 100 | 2.1 ± 2.6 | 123.8 | 4 | 2520 |

[+]For the population data, the average and standard deviation of the ratio from different positions were obtained. The global COV is the ratio of the standard deviation to the average from the previous step.

**Diffusion maps at different positions are provided in Supplementary Figs. 2D-F. Sample data refers to the diffusion coefficients at one particular position for the alginate bead.

*The error associated with the ratio between the diffusion coefficients in the exterior to the interior in the sample data was obtained by performing an error propagation to the errors associated with the diffusion coefficient in the exterior and the interior of the bead. [@]Number of measurements within an area, [&]The total number of autocorrelation from $N_{pos}$ regions is shown here.

The microporous bead, which acts as a physical barrier, led to a lowering of the concentration inside the bead. The effective concentration inside the bead was lower than that of the threshold leading to a decrease in amplitude of the autocorrelation function as seen in Supplementary Figs. 2 G and H.

The diffusion of 20 kDa and 150 kDa neutral dextran molecules is retarded in the interior of alginate beads and biofilms. When the size of the tracer approached the pore size of the system being probed, the tracer exhibited trapped diffusion in Monte Carlo simulations[4], colloidal crystals[5] and agarose gels[6]. Yeon et al. showed that the diffusion coefficient of the molecule decreased with increasing pore radius of the colloids[5]. Similar to the observations above, the ratio between the diffusion coefficients exterior to that of the interior increased for 150 kDa TRITC-dextran when compared to that of 20 kDa TRITC-dextan. For various positions in the bead, the diffusion maps do not show considerable heterogeneity for 20 and 150 kDa TRITC-dextran. In contrast, the diffusion maps of 2 MDa TRITC-dextran show considerable heterogeneity among one another indicating that the pore size of the bead is not uniform across different positions of the bead. This is also evident in the autocorrelation function which differ considerably in the signal to noise ratio at different positions as shown in Supplementary Figs.

2 I and J. Fatin-Rouge et al.[6] showed that for probes that have a comparable size compared to the pore size of the agarose gel, the diffusion coefficients become position dependent. Collectively, these results suggest that the pore size of alginate bead is in between that of the Stokes' radii of 150 kDa TRITC-dextan and 2 MDa TRITC-dextran, which are 7.9 and 27 nm respectively. This indicates that the alginate beads used in this study are less porous than the biofilms. In the experiments described in the main text, the biofilms are estimated to have a porosity larger than 27 nm. The diffusion of solutes in alginate beads is described by the obstruction model of diffusion in hydrogels[7,8]. In this model, the polymer chains of the hydrogel act as a sieve, and solutes with sizes smaller than the pores in the hydrogel can diffuse into the hydrogel. The effective diffusion coefficient inside the hydrogel is a product of the diffusion coefficient in solution with the ratio of the void fraction of the hydrogel to the tortuosity of the hydrogel. In this study, the observed diffusion in alginate beads is consistent with the obstruction model of diffusion of solutes in a hydrogel.

**SPIM-FCS experiments to probe diffusion of charged molecules on biofilm mimics**

Having established that the 150 kDa dextran molecule penetrates into alginate beads, FITC labelled molecules of the same size with positive (diethyl aminoethyl-DEAE) and negative charges (carboxy methyl-CM) were added to alginate beads to study how charge affects the diffusion of molecules in alginate beads. The alginate bead is at the left side of the images in Supplementary Fig. 3. Unlike Fig. 3 in the main text, the positively charged probe does not partition preferentially within the alginate bead indicating that alginate is not the cause of preferential partitioning in biofilms.

**Supplementary Table 4: Diffusion coefficient of FITC labelled positive and negatively charged dextran molecules diffusing in alginate beads**

| Charge | Exterior | | | Interior | | | Ratio* | N* |
|---|---|---|---|---|---|---|---|---|
| | $D$ [μm$^2$s$^{-1}$] | $D_2$ [μm$^2$s$^{-1}$] | $F_2$ | $D$ [μm$^2$s$^{-1}$] | $D_2$ [μm$^2$s$^{-1}$] | $F_2$ | | |
| **Positive** | 63 ± 10 | 0.2 ± 0.1 | 0.3 ± 0.2 | 43 ± 22 | 1.1 ± 1.5 | 0.4 ± 0.2 | 1.5 ± 0.8 | 3 |
| **Negative** | 28 ± 15 | - | - | 29 ± 15 | - | - | 1.0 ± 0.7 | 6 |

$D$ is the diffusion component of the fast moving particle among the two particles in a two-component fit. $D_2$ is the diffusion coefficient of the slow moving particle among the two particles. $F_2$ is the fraction of the second particle.

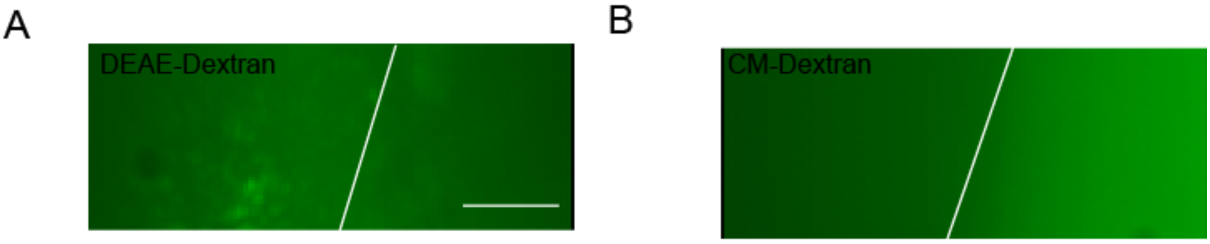


***Supplementary Figure 3****: Fluorescence images of bead to which fluorescently labeled 150 kDa DEAE and CM-dextran-FITC were added. The scale bar measures 10 µm. The bead is positioned to the left in A and B.*

Unlike the data shown for 150 kDa neutral molecules diffusing in alginate beads in Supplementary Table 3, the positively charged 150 kDa FITC-DEAE-dextran exhibits a two-component diffusion profile (Supplementary Table 4). The diffusion coefficient inside the bead is lower than that outside. Unlike 150 kDa-FITC-DEAE-dextran, the negatively charged 150 kDa FITC-CM-dextran does not exhibit a two component diffusion profile. The exterior of the image is brighter than that of the interior in the case of 150 kDa FITC-CM-dextran. In the case of both 150 kDa FITC-DEAE-dextran and 150 kDa FITC-CM-dextran, the diffusion coefficient inside and outside the bead are not significantly different from each other (Supplementary Table 4). This indicates that the charged molecules are not considerably slowed down in the interior of the alginate bead. The experiments on alginate beads validate that Imaging FCS is capable of distinguishing inhomogeneous environments. The diffusion maps obtained can clearly distinguish regions inside and outside the bead.

## Supplementary note 3. Comparison of Imaging FCS measurements with Confocal FCS measurements

The results obtained from Imaging FCS in probing inhomogeneous environments were compared with those obtained from confocal FCS which is a well-established technique.

### Confocal FCS experiments to probe diffusion of neutral molecules on biofilm mimics

In the case of 2 MDa TRITC-dextran, one can clearly distinguish the periphery as seen in the inset of Supplementary Fig. 4C whereas the periphery of the bead is not visible clearly in the case of 20 kDa TRITC-dextran and 150 kDa TRITC-dextran. The noise level in the raw data of the autocorrelation curve in the interior when compared to the exterior for 150 kDa TRITC-dextran is larger than that of 20 kDa TRITC-dextran as seen in Supplementary Figs. 4B and C. This is due to the ease of penetration of a small molecule when compared to that of a large molecule. The diffusion coefficient inside the bead is less than that of the exterior as measured by both confocal FCS and SPIM-FCS in the case of 20 and 150 kDa TRITC-dextran molecules. The ratio of diffusion coefficients between the exterior to the interior for 150 kDa TRITC-dextran is larger than 20 kDa TRITC-dextran as seen in Supplementary Table 5 as well.

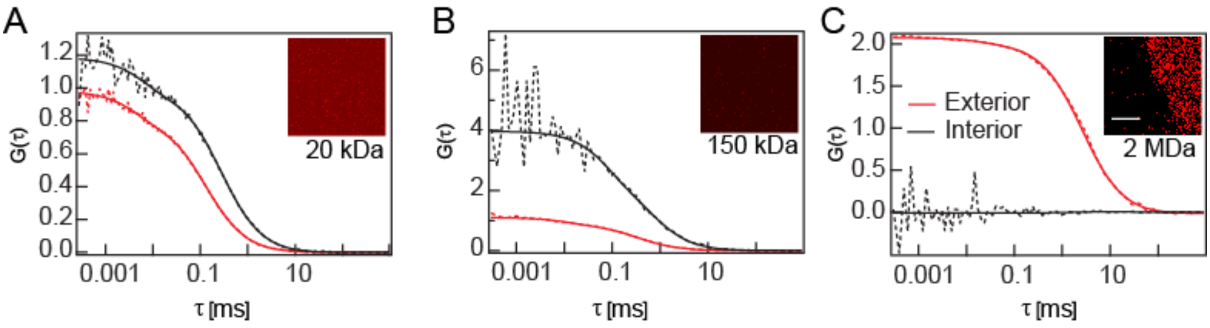


***Supplementary Figure 4:*** *The insets show the confocal images of the bead. The periphery is not visible in the case of 20 kDa TRITC and 150 kDa TRITC. It is clearly visible in the case of 2 MDa TRITC. The autocorrelation curves of 20 kDa, 150 kDa and 2 MDa is shown in A, B and C respectively. The scale bar in C represents 20 µm.*

The autocorrelation curve for 2 MDa TRITC-dextran (shown in Supplementary Fig. 4C) was close to zero when measured at the interior of the bead and hence it could not be fitted. This indicates a lack of penetration into the bead. Exclusion of the 2 MDa TRITC-dextran molecule has also been observed in *S. mutans* biofilms[9]. Yeon et al. showed that the diffusion coefficient of the molecule varied with the pore radius of the colloids and when the size of the molecule was similar to the size of the pore, the molecule becomes entrapped and no correlation could be measured for 40 and 155 kDa dextrans diffusing in 200 nm cavities in colloidal crystals[5]. This suggests that the alginate bead acts as a sieve and does not allow the penetration of molecules larger than its pore size.

**Supplementary Table 5. Diffusion coefficients of TRITC labelled molecules in alginate beads measured by confocal FCS.**

| Size | Exterior [$\mu m^2 s^{-1}$] | Interior [$\mu m^2 s^{-1}$] | Ratio* | N |
|---|---|---|---|---|
| **20 kDa** | 46 ± 13 | 37 ± 7 | 1.2 ± 0.4 | 15 |
| **150 kDa** | 32 ± 1 | 19 ± 3 | 1.7 ± 0.3 | 10 |
| **2 MDa** | 2.4 ± 0.5 | - | - | 10 |

The observation volume of 1x1 bin corresponds to ~3 µL in Imaging FCS while confocal FCS probes at ~0.22 µL. As the binning size is increased, the quality of the autocorrelation increases due to an improved signal to noise ratio and the autocorrelation curves can be fitted. The data in Imaging FCS is reported at 3x3 bin. This is illustrated in Supplementary Fig. 5 for 2 MDa TRITC-dextran molecule. Similar to that of the autocorrelation curve obtained from the confocal FCS as shown in Supplementary Fig. 3C, the autocorrelation curve obtained from 1x1 bin has a poor signal to noise ratio. There is a clear improvement in signal to noise ratio with increase in bin size.

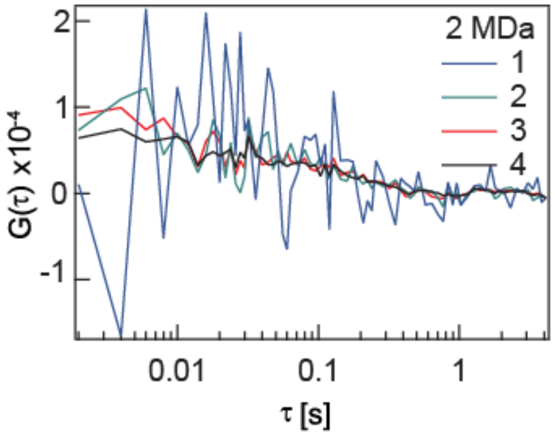


***Supplementary Figure 5:*** *The evolution of autocorrelation functions for increase in bin sizes from 1 to 4 for 2 MDa dextran-TRITC.*

**Comparison of results between Imaging FCS and Confocal FCS**

The experiments with confocal FCS reiterate the fact that the porosity of the measured alginate beads is in between 7.9 and 27 nm. This suggests that estimates from Imaging FCS are reliable and are comparable with those obtained from confocal FCS. In the case of Imaging FCS, 2 MDa had a large heterogeneity in diffusion coefficients whereas in the case of confocal FCS, the autocorrelation curve of 2 MDa TRITC-dextran could not be fitted. These differences are explained based on the difference in observation volumes between Imaging FCS and confocal FCS. Similar to the case of Imaging FCS, the ratio between the diffusion coefficients in the exterior to the interior increases as the probe size increases from 20 kDa TRITC-dextran to 150 kDa TRITC-dextran. Comparison of results between Imaging FCS and confocal FCS validate the use of Imaging FCS to probe inhomogeneous environments.

**Confocal FCS experiments to probe diffusion of charged molecules on biofilm mimics**

Supplementary Figs. 6A and B shows the autocorrelation curves from confocal FCS for the positively charged and the negatively charged molecule. The insets show the confocal images. The fluorescent images obtained from confocal microscope shown in Supplementary Fig. 6B is similar to that from Imaging FCS where the exterior of the bead has a higher intensity when compared to the interior. The autocorrelation curve inside the alginate beads is flat for both the molecules. Hence, the autocorrelation curves could not be fitted. Supplementary Table 6 lists diffusion coefficients only on the exterior of the alginate bead.

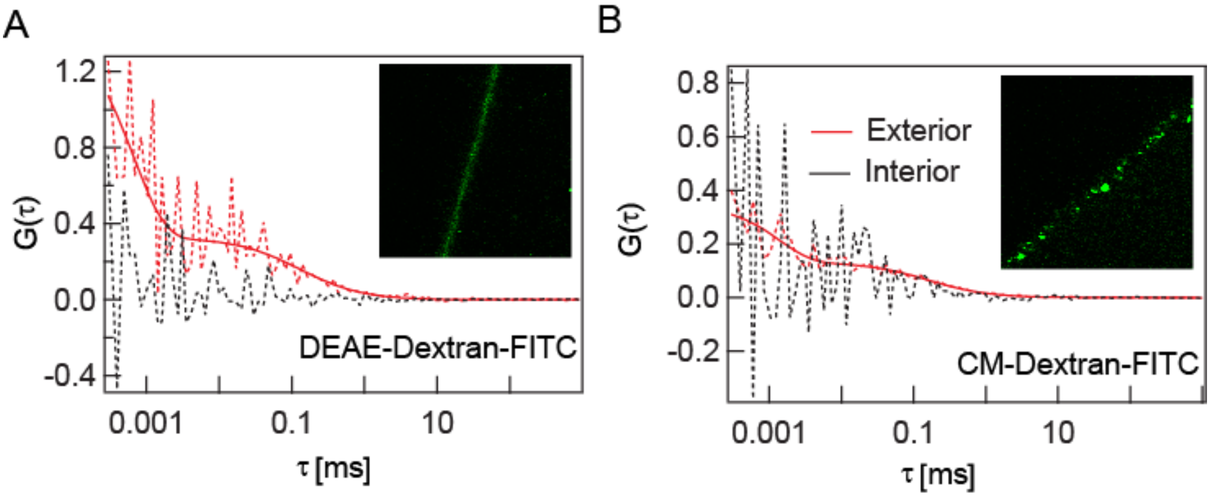


***Supplementary Figure 6:*** *A and B show the fluorescent images of 150 kDa DEAE and CM-dextran obtained from confocal microscopy. The bead is positioned to the left side of the images in A and B. The autocorrelation functions obtained from confocal FCS for DEAE and CM dextran respectively are shown at the bottom of A and B respectively. The insets show the fluorescence images obtained from confocal microscopy.*

**Supplementary Table 6. Diffusion coefficients of FITC labelled charged molecules diffusing in alginate beads from confocal FCS.**

| Size | Exterior [$\mu m^2 s^{-1}$] | Interior [$\mu m^2 s^{-1}$] | Ratio* | N |
|---|---|---|---|---|
| **Positive** | 45 ± 3 | - | - | 10 |
| **Negative** | 55 ± 4 | - | - | 15 |

As explained in Supplementary Fig. 5 for 2 MDa neutral dextran molecule, the autocorrelation curves from SPIM-FCS in the interior of the bead were able to be fitted with theoretical models since the autocorrelation curves are obtained from a larger volume than the confocal microscope. Supplementary Fig. 7 shows the evolution of the autocorrelation curves from SPIM-FCS for the negatively charged CM-dextran. There is an increase in signal to noise ratio in the autocorrelation curves as the bin size is increased from 1 to 4.

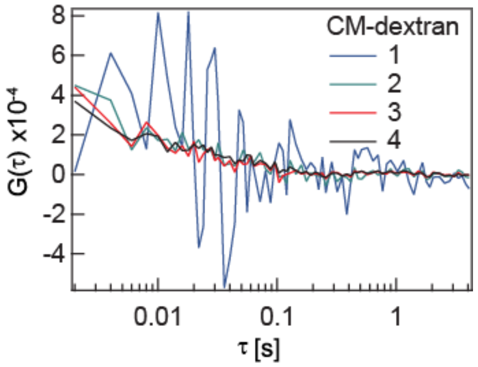


***Supplementary Figure 7:*** *Evolution of autocorrelation curves for 150 kDa CM-dextran-FITC inside the alginate bead for various bin sizes.*

**Other supplementary figures**

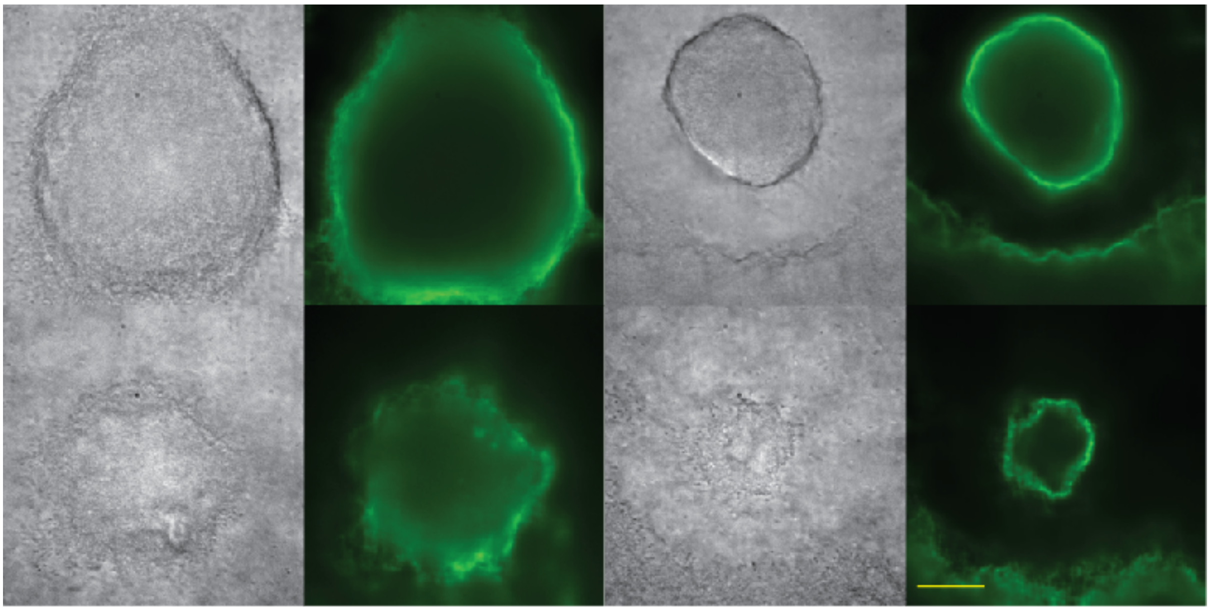

***Supplementary Figure 8:*** *Representative wide field images of 4 microcolonies are shown here. The fluorescence images of the colonies are those stained with fluorescently labeled lectin (ConA- Alexa Fluor 488). The scale bar represents 20 μm.*

**Supplementary notes 4: Simulations**

**Procedure:** 3D random walk simulations of free diffusion of molecules[10,11] were conducted to identify to understand the effects of changes in the number of particles on autocorrelation functions in SPIM-FCS. The concentration of the system was varied by changing the number of molecules diffusing in the system. Two different kinds of simulation were performed, one without noise and one with camera noise. A random number generated using a Gaussian function with a mean of zero and a standard deviation of three was used to add noise to the total amount of emitted fluorescence. 1000 particles with a counts per molecule per second (CPS) of 10000 were simulated for 50000 frames at a time resolution of 0.002. The diffusion coefficient was set to be 10 $\mu m^2 s^{-1}$ with 10 steps per frame.

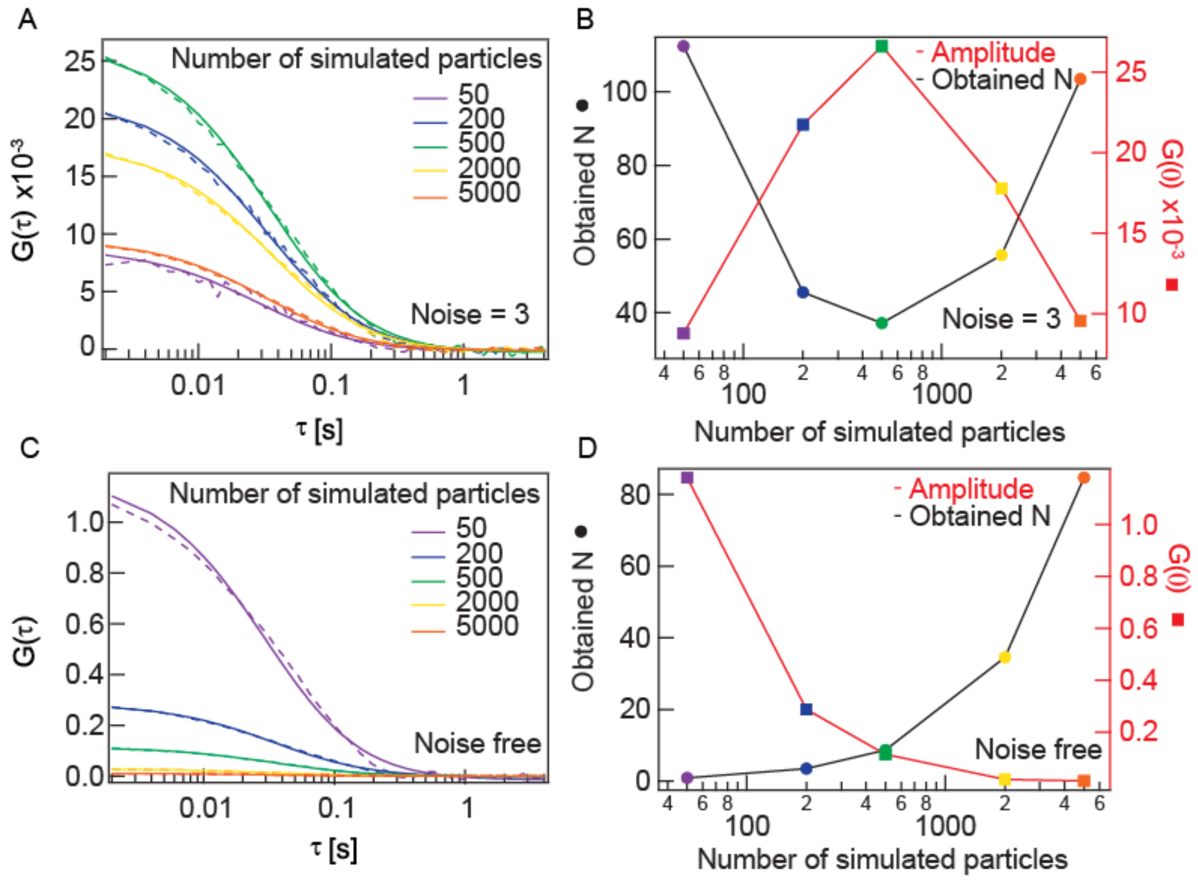


***Supplementary Figure 9:*** *A and C show the autocorrelation curves for different number of particles diffusing in the system for simulations with and without camera noise respectively. B and D show the amplitude and number of particles obtained from fitting the autocorrelation shown in A and C for simulations with and without camera noise respectively. The dashed lines show the raw data while the solid lines show the fit. The amplitude and fitted number of particles for a certain number of simulated particles can be obtained from the markers of the same colour of autocorrelation shown in the right panel. In B and D, the amplitude of the autocorrelation function in displayed in red with the red axis at the right. The fitted number of particles is shown in black with the black axis at the left.*

The autocorrelation function obtained from averaging the 21×21 pixels along with its fit is shown in Supplementary Fig. 9A and C. As expected, there was a reduction in the amplitude of the autocorrelation function with an increase in the number of particles simulated in the

system for noise free simulations as shown in Supplementary Fig. 9D. The number of particles estimated per pixel by fitting the autocorrelation function increased with an increase in the number of simulated particles in the system as shown by the black line in Supplementary Fig. 9D.

Unlike the autocorrelation functions shown in Supplementary Fig. 9C, the amplitude of the autocorrelation did not decrease with an increase in the number of particles for simulation where camera noise has been incorporated. The amplitude increased with an increase in the number of simulated particles up to a certain threshold after which there was a decrease. The inverse relationship between amplitude and the number of particles is maintained even in simulations where noise has been incorporated throughout the entire range of simulated particles varying from 50 to 5000. The monotonic increase in fitted number of particles with the increase in number of simulated particles as seen in Supplementary Fig. 9D is not seen in Supplementary Fig. 9B. The obtained number of particles is erroneously overestimated up to the threshold beyond which the monotonic increase in obtained N with increase in number of simulated particles is regained. Typically, SPIM-FCS is performed in a region where the number of particles is larger than the threshold. The interested reader is referred here for a theoretical treatment on the noise in FCS[12]. In the case of alginate bead and biofilm experiments, the concentration of the fluorophore in the exterior of the bead was chosen in such a way that it was larger than the necessary threshold concentration.

**Other supplementary figures**

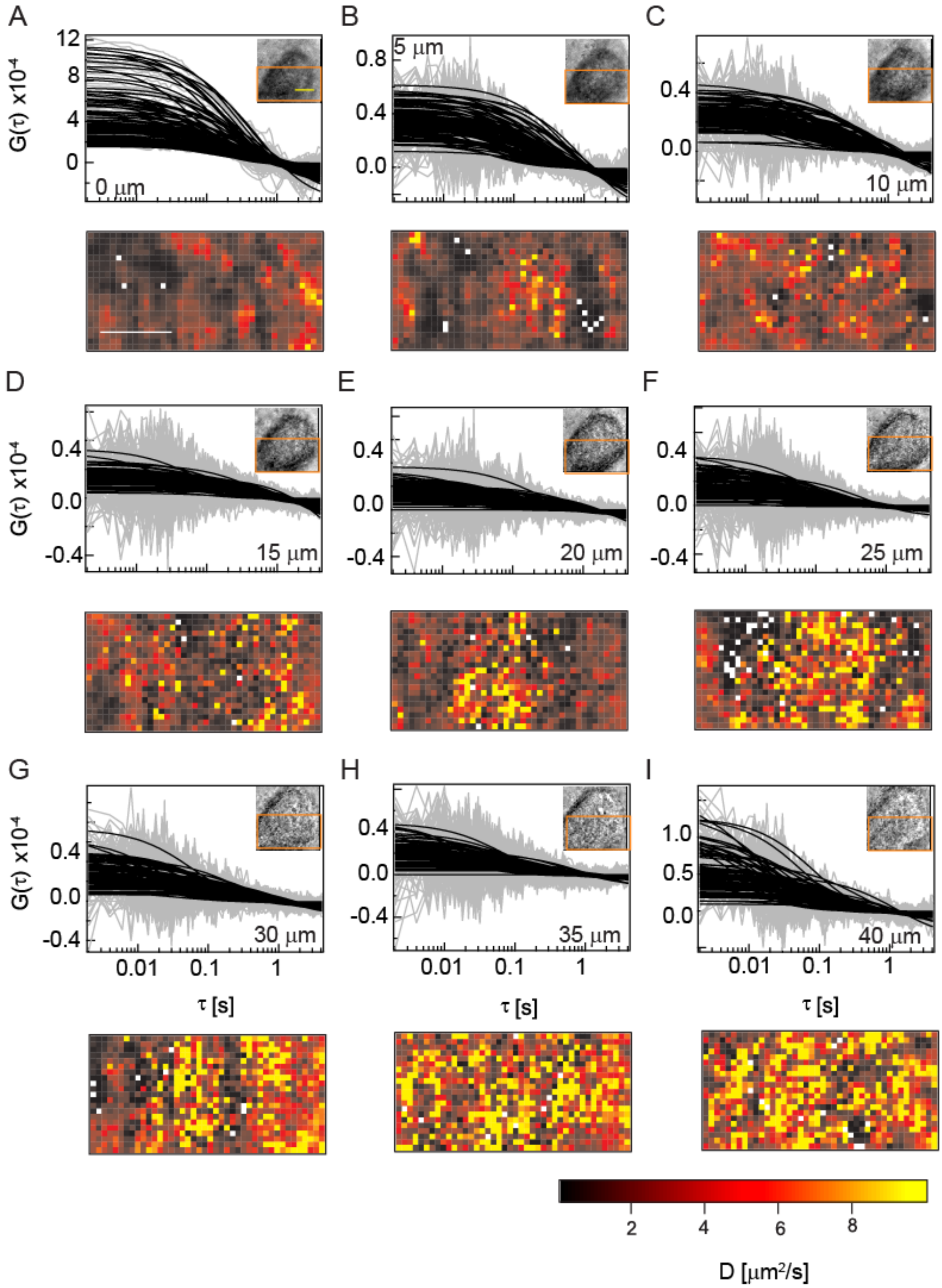


***Supplementary Figure 10:*** *Autocorrelation curves of 2 MDa TRITC-dextran collected at different depths of a microcolony measuring 40 µm at the base. This is an accompany figure for Fig. 2 in the main text. The scale bar in the inset in A and in the diffusion map is 10 µm.*

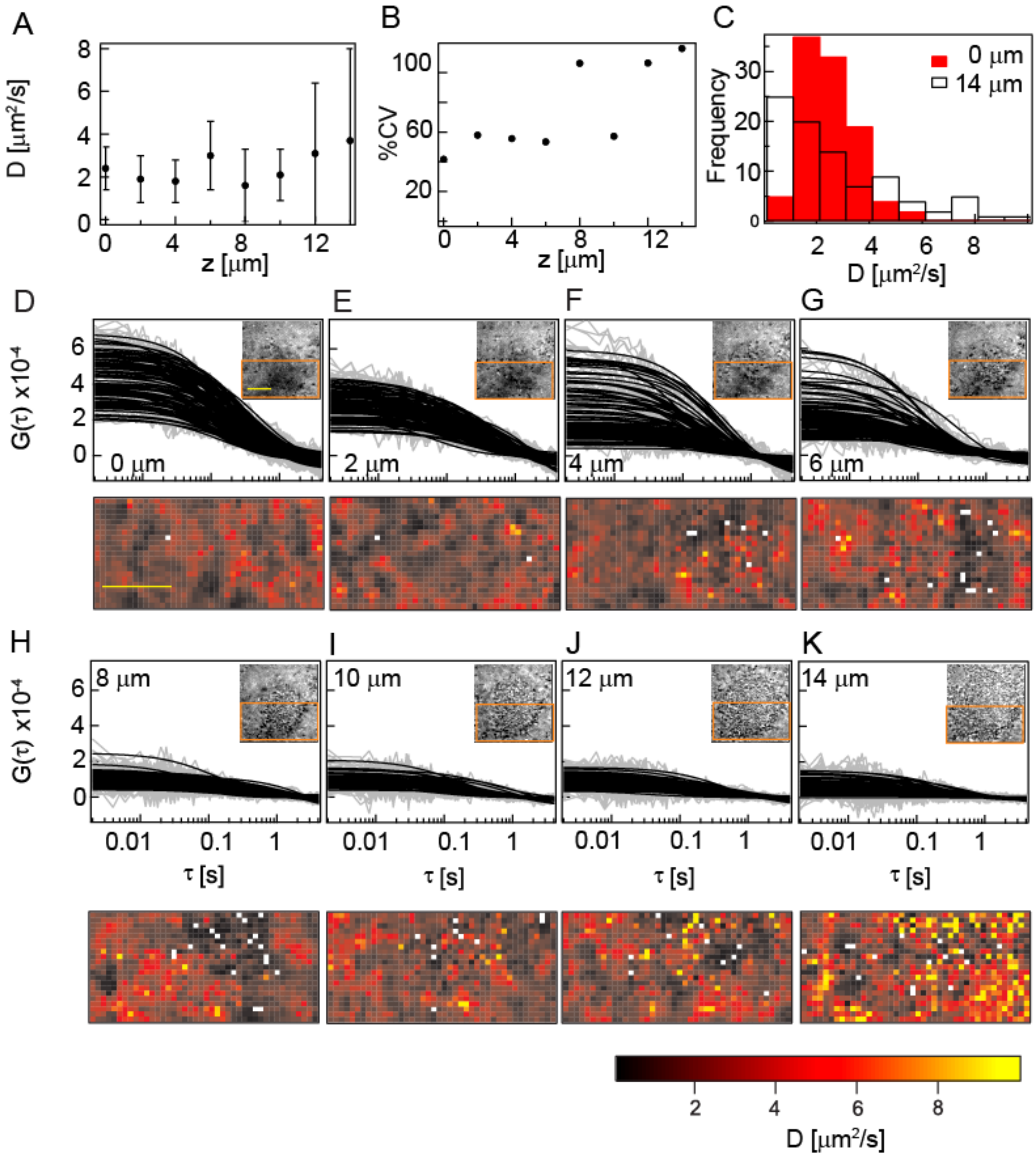


***Supplementary Figure 11:*** *A is a plot of the average diffusion coefficient of 2 MDa TRITC-dextran with depth of the biofilm. The coefficient of variation, which is the ratio of the standard deviation to the mean, is shown in B. C shows the distribution of diffusion coefficients for the solution and at the base of the biofilm. Wide field images, autocorrelation curves and diffusion maps are shown in D-K. The scale bar in the inset and the diffusion map in D is 10 µm.*

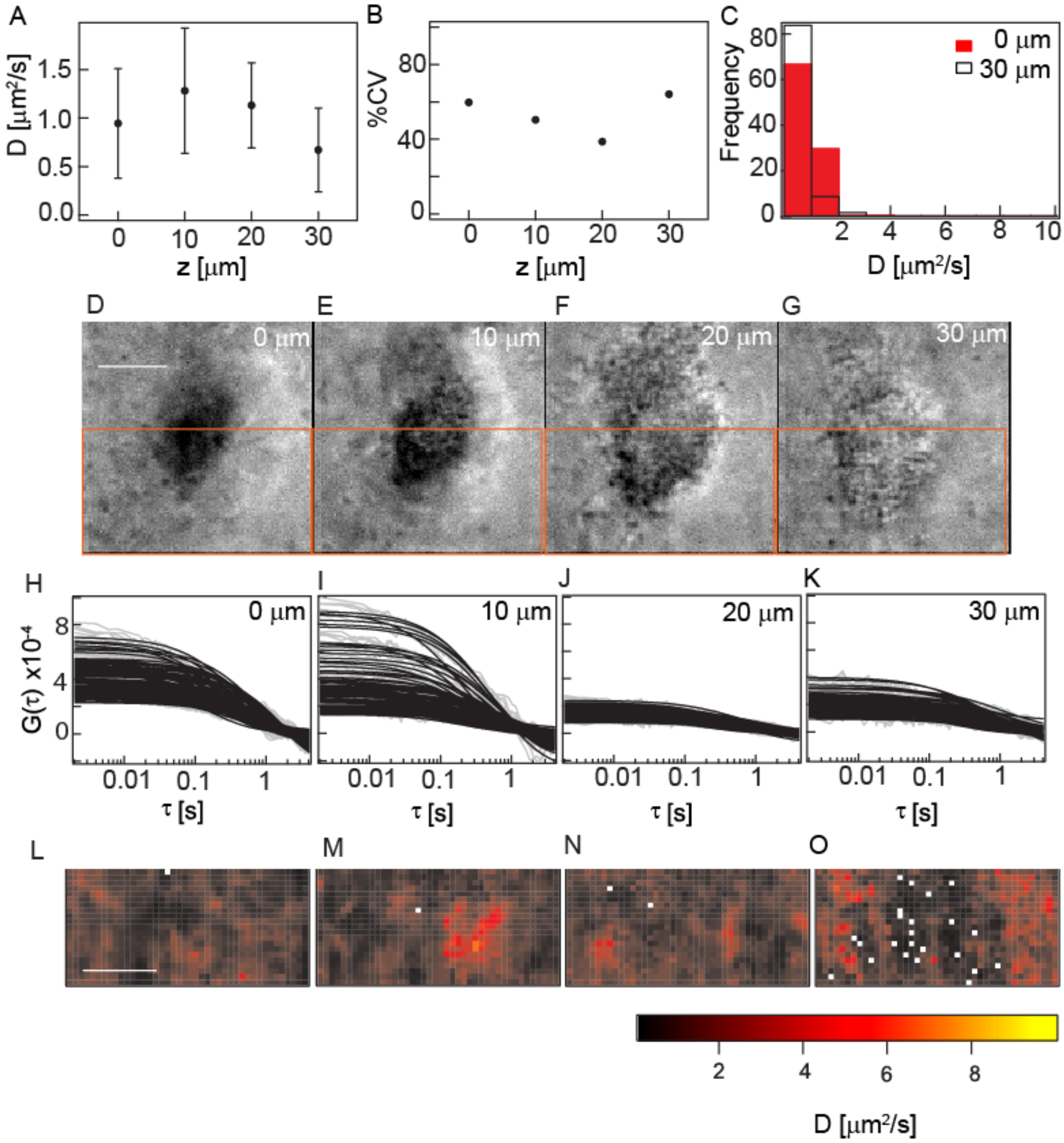


***Supplementary Figure 12:*** *A is a plot of the average diffusion coefficient of 2 MDa-TRITC-dextran with depth of the microcolony measuring 20 µm. The coefficient of variation, which is the ratio of the standard deviation to the mean, is shown in B. C shows the distribution of diffusion coefficients for the solution and at the base of the biofilm. Wide field images, are shown in D-G. The corresponding autocorrelation curves are shown in H-K. The diffusion maps are shown in L-O. The scale in D and L measure 10 µm.*

**Supplementary Table S7. Diffusion coefficients of 150 kDa Dextran-FITC molecules in solution and in biofilms.**

| | | ***D*** **[μm²s⁻¹]** | $\boldsymbol{F_2}$ | $\boldsymbol{D_2}$ **[μm²s⁻¹]** | **No of measure-ments*** | **No of micro-colonies** |
|---|---|---|---|---|---|---|
| **Solution** | **Untreated** | 11.6 ± 5.6 | 0.3 ± 0.2 | 0.5 ± 0.3 | 7 | - |
| | **DEAE** | 15.9 ± 4.9 | 0.4 ± 0.1 | 0.9 ± 0.2 | 4 | - |
| | **CM** | 8.3 ± 0.9 | 0.1 ± 0.1 | 0.1 ± 0.1 | 4 | - |
| **Biofilm** | **Untreated** | 15.1 ± 15.6 | 0.5 ± 0.2 | 0.4 ± 0.5 | 27 | 8 |
| | **DEAE** | 6 ± 4.5 | 0.6 ± 0.2 | 0.4 ± 0.5 | 6 | 5 |
| | **CM** | 5.9 ± 2.9 | 0.6 ± 0.2 | 0.1 ± 0.1 | 6 | 3 |

$D$ is the diffusion component of the fast moving particle among the two particles in a two-component fit. $D_2$ is the diffusion coefficient of the slow moving particle among the two particles. $F_2$ is the fraction of the second particle. Each measurement is an average of 100 diffusion coefficient estimates from a square area.

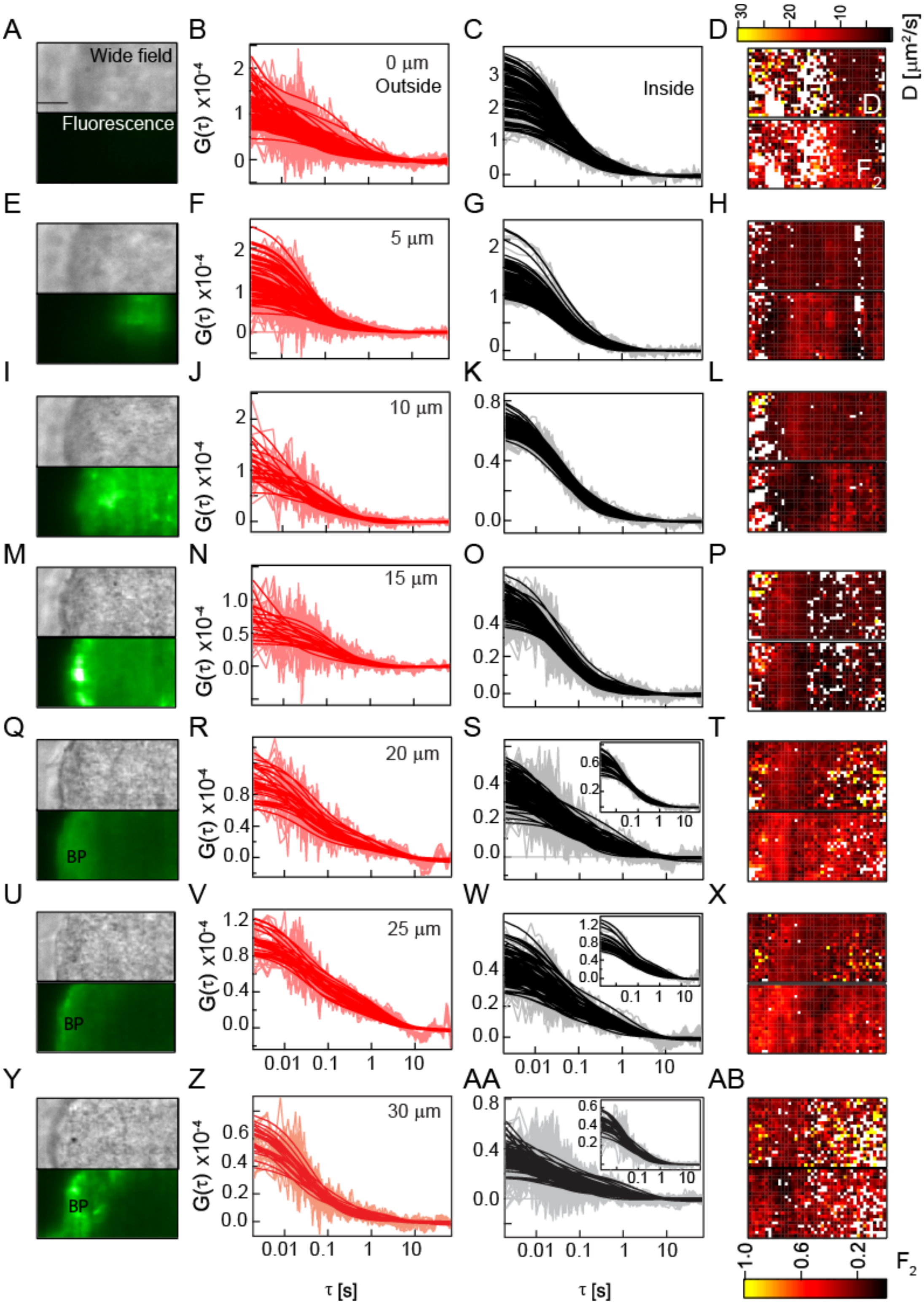


***Supplementary Figure 13:*** *The wide field and fluorescent images of 150 kDa DEAE dextran FITC are shown in the first column. The autocorrelations outside the biofilm are shown in the second column. The third column shows the autocorrelation inside the biofilm. A 2-component fit was performed. The parametric map of the larger diffusion coefficient and the fraction of the second particle are shown in the last column. The autocorrelation at the bright peripheral region (marked as BP in the fluorescent image) is shown in the inset of the S, W and AA. The scale bar in A measures 10 µm.*

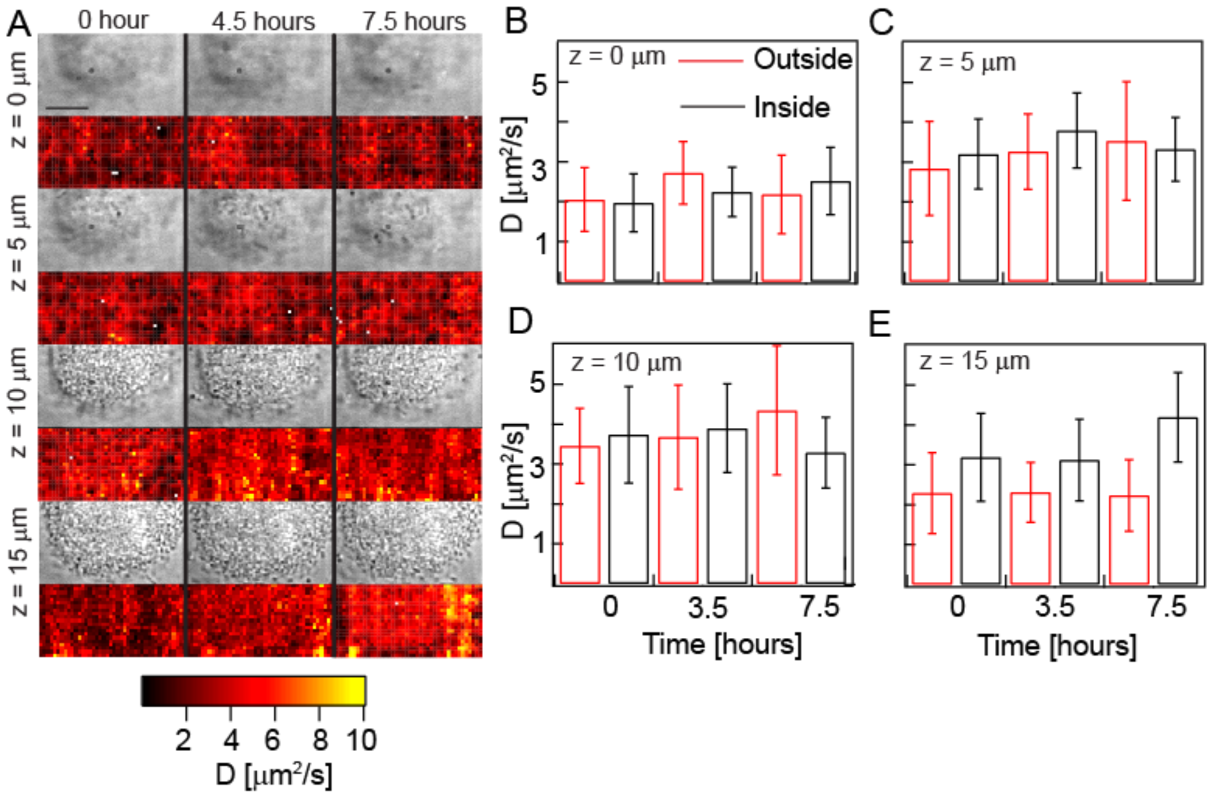


***Supplementary Figure 14:*** *A is a montage of the z stack and time-lapse of the wide field and parametric diffusion maps. The diffusion coefficients of 2 MDa TRITC-dextran in P. aeruginosa PA01 eGFP pBAD yhjH biofilm at different depths and at different time points are quantified and shown in B-E. The scale bar in A is 10 µm.*

## Supplementary methods

### Con A staining

300 µl of 100 µg $mL^{-1}$ of ConA- Alexa Fluor 488 (Cat# C11252, Thermo Fisher scientific) was used to stain the EPS. The dye was injected into the FEP tube and the flow was stopped for 1 hour for the binding. Unbound dye was later washed by flowing medium into the FEP tube.

### Preparation of alginate bead and sample preparation for confocal FCS and Imaging FCS

A 2% alginate solution was added dropwise to 100 mM solution of $CaCl_2$ to form beads. The alginate beads were then rinsed with deionised water 3 times. After rinsing, Phosphate Buffered Saline (PBS) was added to the beads. Only spherical beads (diameter 2-4 mm) were selected for spectroscopic investigation. Selected alginate beads were placed on a glass cover slip for confocal FCS or inside a FEP bag for SPIM-FCS. Appropriate solution of fluorophore was added to the alginate bead and allowed to equilibrate for 1 min. The bead was positioned in such a way that the field of view of the EMCCD camera illuminated by the thinnest part of the light sheet captured the periphery of the bead along with its exterior solution. As control measurements, we performed SPIM-FCS experiments on biofilm mimics using an existing homebuilt set up[1-3]. Fluorescently labelled dextrans of various molecular weights were added to alginate hydrogel beads and SPIM-FCS measurements were made at the interface between the bead and the exterior. Alginate beads were transferred to heat sealed bags made of FEP for imaging.

### Confocal FCS Instrumentation

An Olympus FV1200 Laser Scanning Microscope (IX83; Olympus, Tokyo, Japan) with a PicoQuant upgrade (Microtime 200; PicoQuant, GmbH, Berlin, Germany) was used to perform confocal FCS. A single point laser was focused on a specific point within the alginate bead sample using a water immersion objective. A 50/50 beam splitter was used to split the fluorescence signal into two channels. Cross-correlations between signals in two different channels were performed in order to remove the afterpulsing effect. For Fluorescein Isothiocyanate (FITC) labelled samples, two 513/17 emission filters were used with a 488 nm laser line at 24-48 µW laser power. For TRITC-labelled samples, 600/50 emission filters were used. The measurement time for all readings was 1 min. The FCS curves were then fitted using PicoQuant Symphotime software. The diffusion coefficient of Atto 488 and Atto 565 used for calibration were 400 $\mu m^2$ $s^{-1}$ and 426 $\mu m^2$ $s^{-1}$ respectively. All images were processed using Fluoview Viewer (Olympus, Tokyo, Japan).

### Determination of light sheet thickness

The thickness of the light sheets were measured by placing a mirror at 45° to the plane of the light sheet[13]. The thickness for the various laser lines are shown in Table S8. The Gaussian profile of the light sheet is shown in Supplementary Fig. 13A. The light sheet thickness reported here is the $e^{-2}$ radius of the intensity where the intensity has reduced to 13% of the peak intensity. This value is twice the value of the standard deviation of the Gaussian function used to fit the intensity profile.

**Determination of PSF in xy direction**

The PSF in the x-y direction was measured according to the method described here[14]. When the variation of diffusion coefficient with bin size is plotted, for $PSF_{xy}$ greater than the $PSF_{xy}$ of the system, negative slopes are obtained. In the same plot, for the case of $PSF_{xy}$ less than the $PSF_{xy}$ of the system, positive slopes are obtained. The $PSF_{xy}$, which yields a zero slope, is the $PSF_{xy}$ of the system.

$$PSF = \frac{PSF_{XY}\lambda}{NA} \qquad \text{Supplementary Eq. 3}$$

Where $PSF_{xy}$ is the value at which a zero slope was obtained in the PSF plot, $\lambda$ is the wavelength of light and NA is the numerical aperture of the objective.

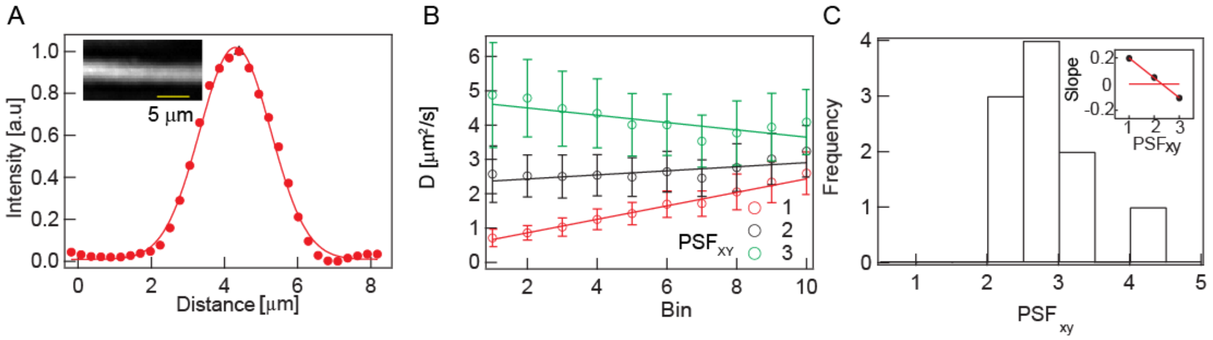


***Supplementary Figure 15**. Instrumental characterization of the two light sheet systems. A) The profile of the cross-section of the light sheet. The intensity shown in dots has been fitted with a Gaussian function to determine the thickness. The insets show the intensity images of the light sheet. B) A calibration plot to determine the PSF in the lateral direction. The dependence of the diffusion coefficient on the bin size and their linear fit for three different set values of $PSF_{xy}$. The zero crossing of the linear fit between the slopes obtained from B and the $PSF_{xy}$ is the PSF of the system. C) The distribution of the $PSF_{xy}$ obtained from PSF plots of molecules ranging from 4 kDa to 2 MDa. The inset shows a representative example of zero crossing of the slope vs $PSF_{xy}$ plot.*

**Supplementary Table 8. Point spread functions in the lateral and axial direction for light sheet – 1.**

| | Wavelength [nm] | $PSF_{xy}$ [µm] | $PSF_z$ [µm] |
|---|---|---|---|
| **Lightsheet1-Camera1** | 488 | 0.7 | 1.4 |
| | 561 | 1.5 | 1.9 |
| | 635 | 1.9 | 6.3 |
| **Lightsheet1-Camera2** | 561 | 0.8 | 1.9 |

The PSF plots were generated for a variety of molecules ranging in molecular weight from 4 kDa TRITC-dextran to 2 MDa TRITC-dextran. A fit to a line was performed for every $PSF_{xy}$. The slopes obtained from the linear fits were then fitted to a line in order to determine the zero

crossing of the x-axis. The zero crossing along the x-axis is the $PSF_{xy}$ for which the diffusion coefficient is a constant for the various bin sizes measured. The average value of this distribution was chosen as the $PSF_{xy}$ of the system. A representative PSF plot is shown in Supplementary Fig. 15B. The distribution of PSFs obtained from size range of 4 kDa to 2 MDa is shown in Supplementary Fig. 15C. One example of zero crossing is shown in Supplementary Fig. 15C inset.

**Custom built SPIM-FCS instrumentation**

The SPIM-FCS measurements on alginate beads were performed on a home built light sheet set up described here. This set up is referred to as light sheet - 2. A detailed description of optics in the home built set up is described here[1-3]. 488 nm (OBIS 488 nm LX, Coherent Inc, USA) and 561 nm (LMX-561S-25-COL-PP, Oxxius S.A, France) served as excitation sources for probes labelled with FITC and TRITC respectively. The laser light was collimated using optical fibres and later beam expanded. Cylindrical lens (f = 75 mm, Thorlabs Inc, USA) placed after beam expansion led to the creation of the light sheet. SLMPLN 20×/NA 0.25 (Olympus, Japan) was used as the illumination objective.

**Supplementary Table 9. Point spread functions in the lateral and axial direction for light sheet – 2.**

| Wavelength [nm] | $PSF_{xy}$ [µm] | $PSF_z$ [µm] |
|---|---|---|
| 488 | 0.7 | 1.2 |
| 561 | 0.8 | 1.2 |

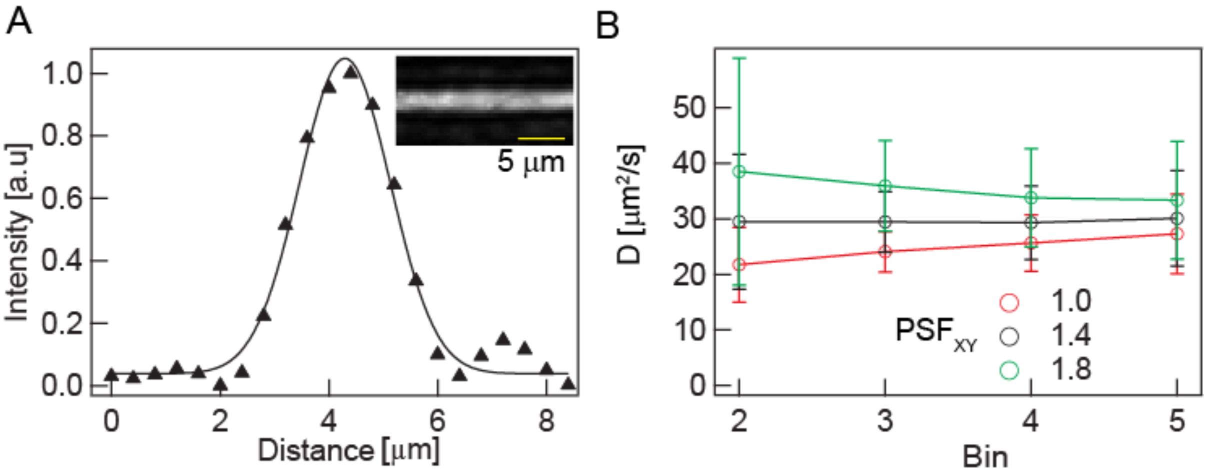


***Supplementary Figure 16****. Instrumental characterization of the two light sheet systems. A) The profile of the cross-section of the light sheet. The intensity shown in dots has been fitted with a Gaussian function to determine the thickness. The insets show the intensity images of the light sheet. B) A calibration plot to determine the PSF in the lateral direction. The dependence of the diffusion coefficient on the bin size.*

The thickness of the light sheet and PSF in the x y direction were measured according to protocols described above and is tabulated below. The PSF calibration plot is shown in

Supplementary Fig. 16. The sample was mounted in a custom-built sample chamber (Physics Mechanical Workshop, NUS, Singapore). LUMPLFLN 60×/NA 1.0W (Olympus, Japan) was used as the detection objective. To remove scattered laser light, a long pass (BLP01-488R-25, Semrock, USA) and a notch filter (NF561-18, Thorlabs Inc, USA) were placed after the detection objective in the case of 488 and 561 nm lasers respectively. The images were acquired using an EMCCD camera (Andor iXon3 860, Andor, UK).

**Supplementary References**


1 Sezgin, E. *et al.* Binding of canonical Wnt ligands to their receptor complexes occurs in ordered plasma membrane environments. *The FEBS Journal*2513-2526 (2017).

2 Singh, A. P. *et al.* The performance of 2D array detectors for light sheet based fluorescence correlation spectroscopy. *Opt Express* **21**, 8652-8668 (2013).

3 Ng, X. W., Teh, C., Korzh, V. & Wohland, T. The Secreted Signaling Protein Wnt3 Is Associated with Membrane Domains In Vivo: A SPIM-FCS Study. *Biophys J* **111**, 418-429 (2016).

4 Netz, P. A. & Dorfmüller, T. Computer simulation studies of anomalous diffusion in gels: Structural properties and probe-size dependence. *The Journal of Chemical Physics* **103**, 9074-9082 (1995).

5 Yeon, W. C., Kannan, B., Wohland, T. & Ng, V. Colloidal crystals from surface-tension-assisted self-assembly: a novel matrix for single-molecule experiments. *Langmuir* **24**, 12142-12149 (2008).

6 Fatin-Rouge, N., Starchev, K. & Buffle, J. Size effects on diffusion processes within agarose gels. *Biophys J* **86**, 2710-2719 (2004).

7 Fiorentino, S. M. *et al.* Membranes in Drug Delivery. (2015).

8 Amsden, B. Solute Diffusion within Hydrogels. Mechanisms and Models. *Macromolecules* **31**, 8382-8395 (1998).

9 Zhang, Z., Nadezhina, E. & Wilkinson, K. J. Quantifying Diffusion in a Biofilm of Streptococcus mutans. *Antimicrob. Agents Chemother.* **55**, 1075-1081 (2011).

10 Veerapathiran, S. & Wohland, T. The imaging FCS diffusion law in the presence of multiple diffusive modes. *Methods* **140-141**, 140-150 (2018).

11 Sankaran, J., Bag, N., Kraut, R. S. & Wohland, T. Accuracy and Precision in Camera-Based Fluorescence Correlation Spectroscopy Measurements. *Analytical Chemistry* **85**, 3948-3954 (2013).

12 Koppel, D. E. Statistical accuracy in fluorescence correlation spectroscopy. *Physical Review A* **10**, 1938-1945 (1974).

13 Krieger, J. W. *et al.* Imaging fluorescence (cross-) correlation spectroscopy in live cells and organisms. *Nature Protocols* **10**, 1948 (2015).

14 Bag, N., Sankaran, J., Paul, A., Kraut, R. S. & Wohland, T. Calibration and Limits of Camera-Based Fluorescence Correlation Spectroscopy: A Supported Lipid Bilayer Study. *ChemPhysChem* **13**, 2784-2794 (2012).
